# What do people fear about cancer? A systematic review and meta‐synthesis of cancer fears in the general population

**DOI:** 10.1002/pon.4287

**Published:** 2016-10-06

**Authors:** Charlotte Vrinten, Lesley M. McGregor, Małgorzata Heinrich, Christian von Wagner, Jo Waller, Jane Wardle, Georgia B. Black

**Affiliations:** ^1^ Department of Epidemiology and Public Health UCL London UK; ^2^ Department of Applied Health Research UCL London UK

**Keywords:** cancer, fear, oncology, screening, worry

## Abstract

**Background:**

Cancer has long inspired fear, but the effect of fear is not well understood; it seems both to facilitate and to deter early diagnosis behaviours. To elucidate fear's behavioural effects, we systematically reviewed and synthesised qualitative literature to explore what people fear about cancer.

**Methods:**

We searched Medline, Embase, PsycInfo, Web of Science, AnthroSource, and Anthrobase for studies on cancer fear in breast, cervical, and colorectal cancer screening and analysed 102 studies from 26 countries using thematic synthesis.

**Results:**

Fears of cancer emanated from a core view of cancer as a vicious, unpredictable, and indestructible enemy, evoking fears about its proximity, the (lack of) strategies to keep it at bay, the personal and social implications of succumbing, and fear of dying from cancer.

**Conclusions:**

This view of cancer as ‘an enemy’ reprises the media's ‘war on cancer’ theme and may affect the acceptance of cancer early detection and prevention messages, since cancer's characteristics influenced whether ‘fight’ or ‘flight’ was considered appropriate.

## 1 BACKGROUND

Cancer has long inspired fear. Despite advances in early diagnosis and treatment of many cancers, a third to half the general population in the United States and United Kingdom say they fear cancer more than any other disease.^1^, ^2^ Population‐based studies have consistently shown that about a quarter to half the population worry to some extent about getting cancer, with 5%–10% experiencing extreme worry.^2^, ^3^, ^4^ On a population level, even these modest percentages equate to a great number of people experiencing significant cancer worry. Fear in itself is unpleasant and burdensome, but it may also affect behaviour, although its behavioural effects are not well understood. Some quantitative studies suggest that it deters help‐seeking and screening for cancer,^5^, ^6^ others that it has a motivating effect,^7^, ^8^ and some find both.^2^, ^9^

These contradictory findings have led some authors to suggest that the effect may depend on the focus of the fear itself.^6^, ^9^, ^10^, ^11^ For example, ‘cancer’ may be associated with perceptions of treatment, incapacitation, and death, and these could be considered separate fears relating to cancer. The discrepant findings of quantitative studies regarding fear's behavioural effects might then be explained by differences in the operationalisation of cancer fear across studies. For example, fear of ‘getting cancer’ may facilitate cancer screening participation to obtain reassurance, while fear about cancer treatments may be a barrier to screening to avoid being diagnosed.^11^ To date, there is no comprehensive overview of the various fears that people may have regarding cancer. Until we understand what it is about cancer that evokes fear, it is difficult to know how best to measure cancer fear, how to allay undue or counterproductive fears, or how to encourage adaptive behaviours in those who may be deterred by their fears.

In the present study, we aim to explore and categorise the fears provoked by cancer that are prevalent in the general, asymptomatic population, through a systematic review and meta‐synthesis of the qualitative literature. We focus on qualitative research because it does not rely on any specific operationalisation of the concept ‘cancer fear’ and allows participants to express their cancer fears in their own words. We use screening as the context because this is the setting in which beliefs about cancer in the healthy, asymptomatic population have usually been explored. To our knowledge, this is the first study to triangulate and synthesise qualitative evidence to create a deeper understanding of the various fears evoked by cancer in the general population, which can be used as a starting point for linking cancer fears to approach or avoidance behaviours.

## 2 METHODS

### 2.1 Definition of cancer fear

‘Cancer fear’ was defined as any fear, anxiety, or worry related to cancer, including causes or consequences of cancer that served as proxies for cancer fear, such as fear of treatment for cancer. Although some authors suggest that ‘cancer fear’ and ‘cancer worry’ are conceptually different,^6^, ^10^ we took an inclusive approach because these distinctions are poorly understood. References to test‐specific fears (eg, pain or cost) were excluded from the analysis because we considered them not specific to cancer.

### 2.2 Scope of search

To explore the cancer fears prevalent in the *general population* (as opposed to fears that may be prompted by possible cancer symptoms in a symptomatic population, or fears prompted by previous experiences or symptoms in a cancer patient population), we limited our review to studies conducted in the context of population screening for breast, cervical, or colorectal cancer (CRC). These are the most widely recommended cancer screenings.

### 2.3 Search strategy and selection criteria

We systematically searched PubMed, EMBASE, PsycInfo, AnthroSource, Anthrobase, and Web of Science from January 1992 until March 2015 (updated from an initial search carried out in June 2013), using the following search terms and Boolean connectors: (cancer OR neoplasm) AND (fear OR worry OR anxiety) AND (screening OR breast OR mammography OR colorectal OR colonoscopy OR FOBt OR occult blood OR sigmoidoscopy OR cervical OR Pap OR cytology). The electronic searches were augmented by hand searching the reference lists of included studies. Inclusion criteria were qualitative or mixed methods studies published in English and digitally available through the London University Libraries (Table 1). Records were screened by one study author (CV) and checked by a second author (MH or LM), with disagreements resolved through discussion. Study quality was not assessed because study quality criteria, such as the CASP checklist,^12^ seemed too dependent on study reporting and did not seem to adequately represent the contribution that studies could make to the generation of a comprehensive overview of cancer fears in the general population. This was due to references to cancer fear in the included studies being infrequent. We therefore took an inclusive approach in order to maximise our dataset and did not use study quality as a selection criterion.

**Table 1:** Inclusion and exclusion criteria

	Rationale/remarks
Inclusion criteria	
Qualitative or mixed methods	Mixed methods only if qualitative part fulfilled criteria
Breast, cervical, or CRC screening	Most universally recommended cancer screenings
Reference(s) to cancer fear in results section or supplementary results files	Primary qualitative data on cancer fear; not just mentioned in introduction or discussion sections
Exclusion criteria	
No cancer fears	For example, fear of test, test cost
No original research article	For example, letters to the editor, reviews
Sample not eligible for screening (or only partially and results for eligible sample not described separately)	For example, ‘key informant’ samples, such as community leaders
Purposive high risk sample, for example, genetic risk	Fears may be different from average risk samples.
Only clinical or self‐breast examination screening methods	No longer considerate adequate methods of breast screening^a^

a Kosters JP, Gotzsche PC. Regular self‐examination or clinical examination for early detection of breast cancer. *Cochrane Database Syst Rev* 2003; **(2)**: CD003373.

### 2.4 Data extraction

Data for each study were extracted to a tabulated pro‐forma with columns for study characteristics and fear‐related findings (including published theme titles, authors' interpretations, and participant quotations) to incorporate the maximum contextual information in our analyses. Study findings which were not about cancer fear according to the definition given above were excluded. Three researchers (CV, MH, and LM) extracted the data in parallel and checked each other's data entry, with disagreements resolved through discussion. During this process, the raw data were read several times by these authors. Only published data were extracted, including supplementary data. The aims of the review, the definition of cancer fear, scope of the search, search strategy, inclusion and exclusion criteria, and data extraction were predefined in a study protocol before the searches were conducted (available from the first author upon request).

### 2.5 Data analysis and meta‐synthesis

We used the thematic synthesis method described by Thomas and Harden.^13^ After familiarising themselves with the dataset, two authors (CV and GB) annotated the extracted data extensively with initial impressions and interpretations, and generated a number of descriptive codes following semantic content. These were grouped into a hierarchy of analytical themes and reconfigured many times in theoretical discussion with each other and the other study authors to establish a coherent structure with constant reference to quotations and authors' interpretations to verify the developing interpretation. This hierarchy was discussed with the whole analysis team (CV, GB, LM, and MH), and this feedback led to some adjustments in two theme interpretations. For the updated literature search (June 2013–March 2015), two authors (CV and GB) annotated the dataset and discussed any new emerging themes. This additional dataset confirmed the robustness of our earlier analysis and identified one new descriptive subtheme, which was cross‐referenced with the earlier dataset (‘fears that talking about cancer causes cancer’ in theme 3). No studies were excluded based on quality. However, our analyses were primarily based on the participant quotations found in the studies and less on the authors' interpretations, which minimises the influence of poorer quality studies' interpretations on our findings. Studies with fewer supporting quotes therefore also contributed less to the synthesis.

## 3 RESULTS

The electronic database searches yielded a total of 5077 results (initial search: 4068, updated: 1009; Figure 1). After screening titles and abstracts, 195 full‐text publications were assessed for eligibility, and 102 studies from 26 countries were included (59.8% North America, 17.6% Europe, 8.8% Middle East, 5.9% Africa, 2.9% Central and South America, 2.9% Oceania, 2.0% Asia). The majority (78%) were published between 2005 and 2015. Data were collected in focus groups (53%), interviews (33%), a combination of these (8%), or a combination of survey and qualitative methods (6%). Reported data analysis methods included content analysis (30.3%), thematic analysis and grounded theory (both 8.8%), and various other methods (13.7%). The analytic methods were described but not specified in 27.5% of studies, and not described at all in 10.8%. Most studies focused on breast screening (37%), followed by cervical screening (26%), colorectal cancer screening (25%), or a combination (12%).

**Figure 1:**
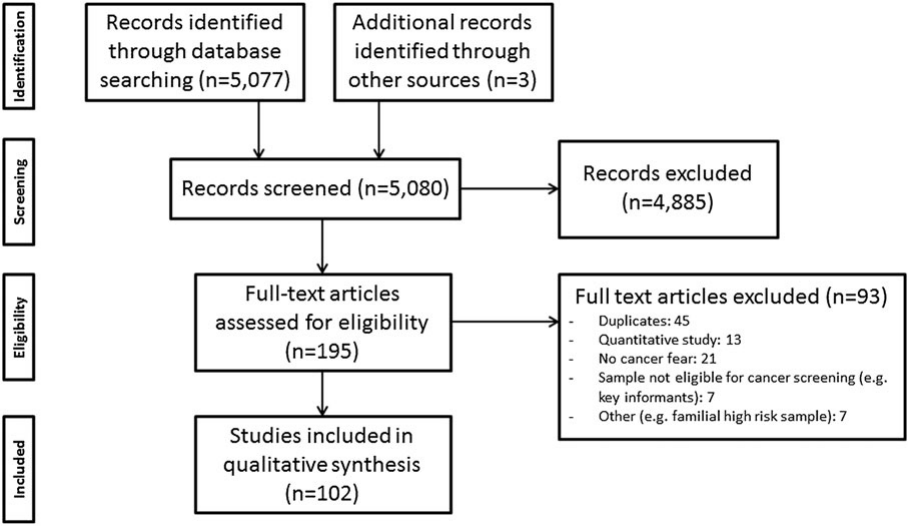
Flow chart of study inclusion

Characteristics of the included studies are presented in Online Supplement 1. All 102 studies together included more than 3500 participants. The majority (74%) only included women, reflecting that two of the three screenings are for women only, but the remaining 27 (>1250 participants) included both genders. A third of all studies (34%) had an ethnically diverse sample of participants, just under a third studied a single ethnic minority group (30%), and the remainder did not specify the sample's ethnicity.

Only two studies explicitly sought to understand the role of cancer fear in cancer screening (Online Supplement 1). Most studies explored factors underlying the decision to participate in cancer screening, some explored cultural meanings and experiences of cancer and screening, and a small number aimed to inform the development of an intervention. In most studies, fear was only one of a number of factors found to influence screening practices, and references to cancer fear in these studies were therefore infrequent. Because of the large number of included studies, this review presents representative rather than complete citations. Full results with complete citations are presented in Online Supplement 2.

## 4 META‐SYNTHESIS

In our interpretation, people's fears were rooted in the meaning of cancer itself as a stealthy, indestructible, and indiscriminate killer,^14^, ^15^, ^16^ which we have labelled ‘cancer as the enemy’ (first theme). This enemy elicits four threat appraisals:

- How close am I to this enemy? (second theme)
- How do I keep the enemy at bay? (third theme)
- What if the enemy attacks? (fourth theme)
- And finally, what if the enemy wins? (fifth theme)

**Figure 2:**
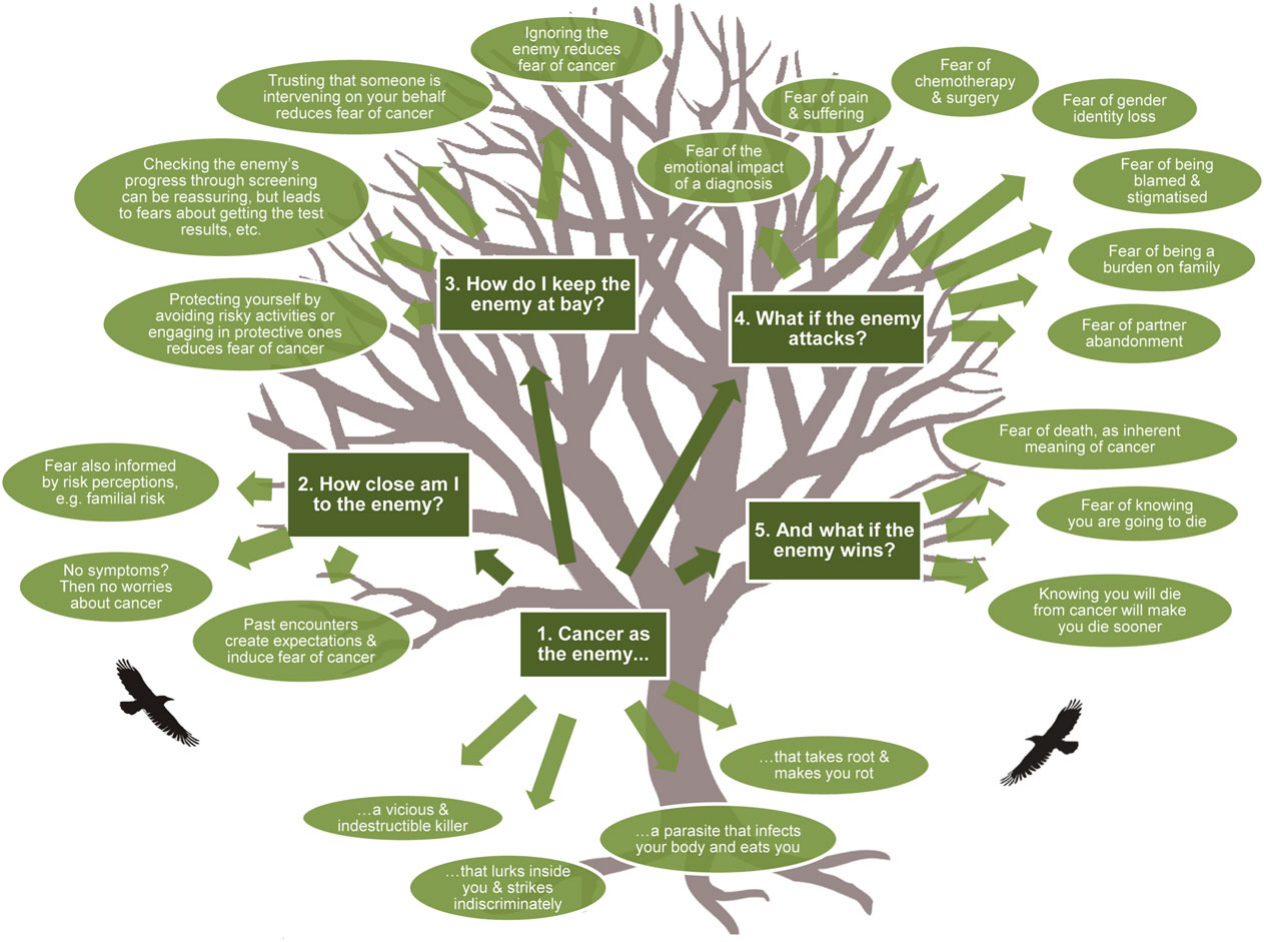
Analytical and subthemes

Figure 2 presents the five analytical themes and subthemes. People's fears do not follow a linear path along the appraisal stages, but fears from different stages can be experienced simultaneously.

## 5 CANCER AS THE ENEMY

In approximately a quarter of all included studies, cancer fear was linked to views of cancer as ‘an enemy’. Participants talked about cancer as if it were not just a disease, but a sentient persona with malicious personality traits, such as viciousness, unpredictability, and indestructibility.^16^, ^17^, ^18^, ^19^ People described cancer as lurking inside you, spreading stealthily and inescapably.^19^, ^20^ By the time it revealed itself, it was believed to be too late to do anything,^18^ which created a sense of betrayal: ‘The cancer is a traitor… You can be examined all the time… and nothing comes up, and then when you find out you have cancer it'*s* too late’*.* 19 Echoing its strong association with death (see theme 5), cancer was seen as an indiscriminate killer,^16^, ^20^, ^21^ that would return endlessly despite treatment.^16^, ^19^, ^22^

Participants in several studies described cancer as a parasitising organism that comes from outside and infects^22^ the body like viruses,^16^ bacteria,^16^ or worms^17^, ^19^, ^23^ and which could be transmitted to others.^16^, ^17^, ^22^ Notions of cancerous growth were strongly related to destructive qualities, such as burning, rotting, or being eaten away.^16^, ^23^, ^24^ The aggressive personality of cancer was a source of much anxiety and fear.^16^, ^19^, ^20^, ^24^

## 6 HOW CLOSE AM I TO THE ENEMY?

In more than half of included studies, participants assessed their own relationship to cancer in terms of proximity. Greater proximity increased fear, and a greater distance reduced fear and created a feeling of safety. Proximity was informed by cancer encounters, the presence of symptoms, and assessments of personal risk.

### 6.1 Cancer encounters

Past encounters with cancer created a series of expectations about cancer.^15^, ^16^, ^19^, ^21^, ^25^, ^26^ Mass media campaigns could allay cancer fear and encourage early detection behaviours^16^ but could also lead to confusion when messages of increased survival ran counter to direct experiences of cancer in others (see theme 5),^19^, ^21^ which mainly seem to shape people's cancer fear. In about a quarter of included studies, participants related their fear to witnessing cancer's consequences in friends and family,^16^, ^19^, ^20^, ^21^, ^24^, ^26^, ^27^, ^28^, ^29^ which ranged from seeing others suffer the effects of chemotherapy and surgery^28^, ^29^ to knowing people who had died from cancer.^16^, ^19^, ^20^, ^26^, ^27^, ^28^ These encounters inspired fear through contemplation of one's own fate: ‘My son's stepmother … was diagnosed with bilateral breast cancer. It brought up a lot of fear for myself because […] I don't know if I could handle that’.^29^ For most people, this fear motivated screening attendance^16^, ^26^, ^27^, ^28^, ^29^: ‘I don't like being without (a mammogram) for a long time. So many people have died of cancer. I'm afraid’*.* 27 For others, it promoted screening avoidance, usually because of coexisting negative beliefs about treatment or survivability,^21^, ^26^ which evoked a ‘I don't want to know it before my time’ mind set.^21^

### 6.2 Symptoms

Symptoms were believed to indicate greater proximity to cancer and therefore inspired fear. In about a quarter of included studies, participants felt reassured by an absence of symptoms, which was seen as an indication that there was no need to attend screening.^14^, ^15^, ^16^, ^17^, ^22^, ^25^, ^28^, ^29^, ^30^, ^31^ A dominant belief was that medical services are only for the sick,^14^, ^22^, ^25^ and only the appearance of symptoms would signal advancing cancer and prompt screening attendance.^14^, ^15^, ^16^, ^17^, ^25^, ^29^, ^30^ Pain, in particular, was seen as an important cue to action^17^, ^30^: ‘If I'm not in pain, I think there's probably no need [for screening]’*.* 17

### 6.3 Assessment of risk

Perceptions of causation, control, and risk of cancer were interrelated factors indicating proximity to cancer. People distinguished causes and risks that were perceived to be under individual control (discussed as part of theme 3) from those that were not. Participants mentioned a number of uncontrollable cancer risks, such as age and pollution,^17^, ^19^ but only familial or genetic factors strongly influenced people's cancer fear.^15^, ^16^, ^20^, ^24^, ^25^, ^26^, ^28^, ^32^ Because familial risk could not be influenced directly, participants tried to find other ways to allay their fears, such as by adjusting their diet^20^ or participating in screening^15^, ^16^, ^25^, ^28^: ‘[…] I do have cancer in my family, and so that is what I'm afraid of. That's one reason I have my Pap smear and my mammogram’.^25^ For some participants, familial risk was such a strong risk indicator that a *negative* family history led them to believe that there was no need to worry about cancer or attend screening^24^: ‘So many women believe that it's hereditary and that if they don't have it in the family, then what's the purpose of doing it [getting a mammogram]?’.^33^

A dozen studies described people who were not afraid of cancer at all, because they did not feel susceptible to it or had never experienced it in anyone close to them. This was linked to a lack of motivation to attend screening.^17^, ^26^, ^34^ In some of these studies,^24^, ^35^ nonwhite ethnicity ameliorated cancer fear because some thought cancer was a ‘Western disease’ to which they were less susceptible, making screening unnecessary.^24^, ^35^ ‘The [women's] attitudes rejecting of screening were based on […] concerns about whether mammograms are as effective for Chinese women as they are for Western women. This is based on their belief that breast cancer is a “Western disease” and that Caucasian women are prone to breast cancer as a result of having larger breasts’.^35^

## 7 HOW DO I KEEP THE ENEMY AT BAY?

Negative emotional states such as fear seldom remain unregulated.^10^ The third theme explores fears and worries associated with four strategies to keep the enemy at bay.

### 7.1 Protecting yourself against cancer

In about a third of included studies, fear was regulated by controlling certain risks. Relatively few studies mentioned lifestyle factors, such as diet and exercise, as a means of controlling risks and allaying fear.^20^, ^24^ Instead, the focus was mainly on other factors that were deemed risky, such as worrying about cancer, and cancer screening.

Some believed that cancer could be the result of worrying, thinking, or talking about cancer.^14^, ^26^ They believed that worrying induced stress, which could facilitate cancer development, and so they worried about being worried about cancer, constituting a form of meta‐worry^14^, ^24^: ‘Just by worrying can we become sick. […] I always worry and that I might get cancer’^14^ Others believed that thinking or talking about cancer was risky,^20^, ^21^, ^22^, ^24^, ^29^, ^36^ ‘fearing that just uttering the word [cancer] would result in getting the disease’^36^ and thus avoided the topic of cancer.

Of the 49 studies about breast cancer screening, more than a quarter mentioned the screening itself as a risky activity that was best avoided because of the radiation^15^, ^16^, ^18^, ^21^, ^25^, ^35^ and compression involved^15^, ^28^, ^35^: ‘I heard that they have to press your breasts until they can fit into the machine. […] What if […] it hurts my breasts and I develop cancer later. Who knows?’*.* 35 Compression during mammography was also linked to fears of a tumour bursting and spreading,^37^ and therefore best avoided. Breastfeeding, on the other hand, was seen to protect against breast cancer, and thus reduced cancer fear.^16^, ^24^

### 7.2 Check the enemy's progress through screening

Screening presents an opportunity to obtain reassurance by checking up on cancer's progress, but nearly a third of included studies described how facing the prospect of this knowledge can (usually temporarily) induce cancer fear. Some reported an intense fear, almost culminating in an expectation, that the test would reveal a cancer diagnosis.^14^, ^29^, ^30^ ‘[I'm afraid of] what they might find. That is my biggest fear […]’*.* 30 Furthermore, a positive test result was often seen as a direct indicator of having cancer, leading to anxiety about the moment the results were received.^19^, ^21^, ^36^, ^37^, ^38^

Having to wait for results prolonged anxiety until the ‘all clear’ was given.^20^, ^30^, ^39^ Other factors that enhanced anxiety about cancer were recalls, only being contacted if further tests were needed (which created prolonged anxiety about having missed the call^39^), and unclear communication of results including benign findings,^18^, ^19^, ^24^, ^30^, ^37^ which put some people off reattending screening, sometimes permanently^18^: ‘The doctor [said] oh it's just calcium deposits—which took 30 years off my life’.^37^ Thus, some people use screening to reduce cancer fear, but the screening test itself is also fear provoking.

### 7.3 Trusting that someone is intervening on your behalf

A third strategy to allay cancer fear is to trust in someone who will guard against cancer on your behalf. In some studies, people delegated this responsibility to their doctor, and lacking a doctor's recommendation for screening was sometimes reassuringly interpreted that there was no need to worry.^17^, ^18^, ^21^ More commonly, however, participants reduced their fear of cancer by looking to God.

Religion was invoked in about a quarter of included studies.^14^, ^15^, ^16^, ^18^, ^19^, ^20^, ^22^, ^23^, ^24^, ^27^, ^30^, ^32^, ^36^, ^38^, ^40^ In some studies, this was only to help cope with fear of cancer,^20^, ^22^, ^27^, ^40^ but the majority of participants also used it to abdicate their own agency over the threat of cancer^14^, ^15^, ^16^, ^18^, ^19^, ^23^, ^25^, ^30^, ^32^, ^36^, ^38^: ‘I don't worry about [breast cancer screening] because only God can decide when it's my time to go.’^30^ These participants saw cancer as part of God's plan or test, and their own role as one of acceptance and patience.^18^, ^30^, ^38^ This view was associated with lower motivation to attend screening.

### 7.4 Ignoring the enemy

Participants in more than half of included studies chose to ignore the existence of cancer altogether, because they were too fearful of being diagnosed. Statements such as ‘ignorance is bliss’,^38^ and ‘what you don't know you don't worry about’^41^ were often used to explain why they did not participate in screening.^16^, ^18^, ^22^, ^23^, ^24^, ^36^, ^37^, ^38^, ^39^, ^41^ They prioritised feeling good or normal over the relative advantages of early detection^19^, ^22^, ^31^, ^32^, ^33^, ^34^, ^36^, ^37^, ^41^ and avoided screening tests because they considered them as taking an unnecessary risk of finding cancer^23^, ^34^ as if spontaneously making it appear: ‘I have this fear that if I check for it and I find something then my life is gonna change. But […] if I don't check, I don't find anything and nothing changes’*.* 34

In about a sixth of included studies, participants ignored the threat of cancer because it competed with more pressing worries, such as other health problems^31^, ^34^ or struggles to meet the demands of everyday life.^21^, ^24^, ^28^, ^31^, ^34^ This was especially prominent in those from poorer backgrounds and developing countries.

## 8 WHAT IF THE ENEMY ATTACKS?

Although all reviewed studies were conducted in healthy populations, many participants expressed fears about being a cancer patient, which could be subdivided into fears about the emotional and physical consequences of a cancer diagnosis, and its social consequences.

### 8.1 Emotional and physical implications of being a cancer patient

Fears about the emotional and physical consequences of a cancer diagnosis were mentioned in more than half of included studies. In a quarter of included studies, participants mentioned fears about how to emotionally ‘handle’ a cancer diagnosis, expecting to feel devastated, anxious, sad, and depressed^14^, ^16^, ^17^, ^18^, ^23^, ^24^, ^29^, ^32^, ^36^, ^38^, ^39^, ^40^ and having their world fall apart: ‘You can go in there [ie, screening] thinking nothing is wrong and come out with your whole life being changed’*.* 32

Fear of the physical implications included fears about the disease course and its treatments. Some were afraid of cancer's spread and feared that it would be too late to stop it by the time it was detected,^15^, ^18^, ^22^, ^29^, ^38^ but fears associated with cancer treatments^15^, ^16^, ^19^, ^20^, ^23^, ^24^, ^25^, ^30^, ^31^, ^36^, ^38^ such as pain, suffering, and bodily changes (including hair loss and resection scars) were more prevalent and were mentioned in more than 40 studies. Chemotherapy^20^, ^36^ and surgery^15^, ^16^, ^20^, ^22^, ^23^, ^24^, ^25^, ^27^, ^28^, ^29^, ^30^, ^38^, ^39^, ^40^, ^41^, ^42^ were particularly fearful, partly stemming from cancer encounters in others (see also theme 2). Fear of surgery was also expressed as a fear of having body parts cut off and deformed, particularly if this would be visible to others^16^, ^20^, ^23^, ^24^, ^25^, ^27^, ^29^, ^41^: ‘[…] I would prefer to die and be buried in one piece than being cut and sold by kilo’*.* 16

In the context of breast and cervical screening, some women expressed fears of losing intimate body parts. The breasts (and to a lesser extent the cervix) were widely considered to be symbols of femininity and motherhood,^16^, ^23^, ^24^, ^28^, ^39^, ^40^, ^42^ and losing these body parts was likened to being ‘less of a woman’^28^:

For many of our informants, the breast was not simply an organ, but a symbol of femininity. Therefore they saw breast cancer as not only threatening their lives, but also their womanhood, since most believed that breast cancer would always lead to the removal of the breasts. […] Because breast cancer was seen as a ‘lady‐killer’, informants were doubly fearful of the disease. Typical comments were ‘If I lose my breasts, I will no longer feel I'm a woman’ and ‘I think breast cancer is the most scary disease for a woman because it takes away the breasts’ [42, breast, Australia].

For some, fear of surgery was enhanced by the belief that cutting cancer causes it to spread^22^, ^30^ and cannot provide a long‐term cure^16^, ^18^, ^19^, ^20^, ^21^, ^22^, ^38^: ‘But you also hear the rumours where people have been opened up and it seemed like when they were opened up, the cancer spread. And I think that is my biggest fear’.^22^ Fears of treatment led to screening avoidance,^15^, ^20^, ^21^, ^22^, ^23^, ^24^, ^31^, ^36^, ^38^, ^41^ although there were some exceptions.^15^, ^29^, ^31^

### 8.2 Social implications of being a cancer patient

Fears about the social consequences of a cancer diagnosis were mentioned in about a third of included studies. Half of these mentioned fears about others' reactions, such as being stigmatised or blamed,^14^, ^16^, ^22^, ^25^, ^26^, ^35^, ^36^, ^40^, ^42^ since cancer was sometimes seen as a punishment for sins or bad karma.^14^, ^42^ Cervical and breast cancer were associated with promiscuous behaviour, provoking fears of social rejection and gossip^42^: ‘[Women] are worried about what will people say, […] questions will be raised and I will have to feel ashamed’*.* 26 Some therefore preferred to avoid screening to avoid being diagnosed.^26^, ^35^, ^36^, ^40^

About a sixth of included studies mentioned fears about the financial, physical, or psychological consequences of a cancer diagnosis on the immediate family^15^, ^16^, ^20^, ^23^, ^24^, ^31^, ^35^, ^36^, ^38^: ‘[A diagnosis of cancer] can be a burden on you… mentally, physically, financially… and a burden on your family when you're so sick’*.* 38 In addition, many women feared partner rejection and abandonment after cancer treatment.^16^, ^24^, ^28^, ^30^, ^36^, ^40^ They felt that cancer treatment, particularly for breast cancer, would leave them incomplete, unattractive, and sexually compromised, which they felt would render them worthless as a wife or mother^16^, ^17^, ^24^, ^28^, ^30^, ^36^: ‘[…] The first thing to come to mind is “Oh, [my husband] is going to get another woman now… I'm no longer attractive”^*30.*^ These fears were linked to preferences for screening avoidance,^24^, ^35^, ^36^, ^38^ although there were again some exceptions.^15^, ^31^

## 9 AND FINALLY, WHAT IF THE ENEMY WINS?

The last theme consists of fears about mortality. ‘Death’ was an inherent meaning of cancer in more than a third of included studies, and this was a source of intense fear^14, 16, 20–25, 28, 30, 36, 38, 39, 42:^ ‘[…] as soon as you hear cancer you think of the ultimate. There*'s* nothing more then, but for this person to die’*.* 39 Some participants were unable to reconcile messages from doctors and the media about improved cancer survival with what they had experienced or feared to be the case, which sometimes led to confusion^19^, ^20^, ^21^: ‘They say that you don't die from cancer and everyone does’*.* 19

Some said that it was better to die without knowing you had cancer,^16^, ^23^, ^24^, ^28^, ^36^ suggesting that fear of dying is also fear of knowing it would happen: ‘I'd rather not go [for screening], that way the doctor can't tell me that I have cancer, because just by knowing, that I will die, […] I would die.[…] From knowing. From the fear’.^24^ This may be related to the ‘ignorance is bliss’–type approach to cancer as described in theme 3. The high prevalence of fears about mortality in general suggests that the association of cancer with death is still very prominent, cross‐cultural and, together with fears about what it is like to be a cancer patient (theme 4), perhaps the most important component of cancer fear.

## 10 DISCUSSION

This meta‐synthesis aimed to explore and categorise cancer fears in the general population. It drew out the multidimensionality of cancer fear, with a view of cancer as an enemy at its core—reprising the ‘war on cancer’ theme that dominates the media.^43^ Cancer fears related to perceptions of proximity; the strategies to keep the enemy at bay; the emotional, physical, and social implications of disease; and dying. We identified factors that enhanced or diminished cancer fear, such as cancer encounters or symptoms, and factors that could become objects of fear in themselves, such as screening and treatment.

People's fear seemed to be rooted in their view of cancer as an enemy, which raises important questions about where this view comes from and the effects it has. The cancer experience became imbued with militarism in the previous century, possibly to secure funding for cancer research.^44^ Today, war and violence metaphors are still often used by cancer charities in fundraising campaigns. The metaphor was also adopted by the mass media^43^ and made its way into the discourse of individual patient experience,^45^ a development that may have been reinforced by the idea that ‘a fighting spirit’ could improve survival.^46^ Although no evidence has been found to support this claim, the fighting metaphor stuck.

The war metaphor for cancer has recently become criticised, however. Patients object to the blame it may to attribute to them for ‘not winning their battle’,^45^ and recent evidence suggests that it may have detrimental effects on some cancer‐prevention behaviours.^47^ Campaigns that portray cancer as an enemy, such as charity fundraising campaigns, may discredit public health messages about prevention and early detection of cancer. This could result in increased cancer worry and scepticism about the preventability of cancer, which are associated with lower adherence to recommendations regarding smoking, exercise, and fruit and vegetable intake, making the metaphor potentially harmful for public health.^48^, ^49^ Indeed, in our meta‐synthesis, we noted an absence of references to controllable lifestyle factors in the risk assessments of those who were fearful, and very little use of behavioural strategies to reduce cancer risk and allay cancer fear, despite current estimates that about 40% of cancers in developed countries are due to lifestyle choices.^50^ Although more research into the causal link between enemy metaphors and diminished cancer prevention behaviours is needed, we caution that campaigns that appeal to the intuitive sense of cancer dread and endorse and exploit people's fear of cancer may impede the adoption of cancer‐preventive health behaviours. Getting rid of mixed messages in the public portrayal of cancer may thus be a key factor for future policies.

Our meta‐synthesis highlighted some other implications for public health: a lack of symptoms, a negative family history of cancer, and lower cancer risk perceptions among some ethnic minority groups all reduced fear of cancer to the point where screening was seen as unnecessary, and these erroneous beliefs may be targets for behaviour change. The finding that symptoms are used as a way to gauge the need for screening in about a quarter of studies is mirrored by quantitative studies that suggest that a quarter to a third of the population feel there is no need to screen in the absence of symptoms.^51^, ^52^ In addition, only a minority of common cancers are associated with a hereditary predisposition, so a negative family history may provide a false sense of security.^53^ Moreover, research shows that some ethnic minority groups actually have a *higher* cancer burden,^54^, ^55^ which is partly attributed to their lower uptake of screening.^56^, ^57^

Implications for future research follow from our finding that ‘cancer fear’ is not a single entity but consists of various interrelated fears that may interact with pre‐existing beliefs to affect behaviour. For example, the belief that a screening test could cause cancer meant that the screening test became a proxy of cancer fear. Cancer fears could also interact with each other. For example, the fear of cancer treatment could outweigh the fear of dying from cancer. This meta‐synthesis has generated tentative findings on the behavioural effects of different cancer fears as they were reported in our qualitative dataset, but these links warrant further evaluation in quantitative studies.

Our study may be limited by the infrequent references to cancer fear in the included studies, but the large number of included studies may have (at least partially) compensated for this. Generalisability may also be limited because data were only collected in the context of breast, cervical, and CRC screening. Future studies will need to examine the robustness of this taxonomy in the wider general population (eg, in less routine screenings, such as PSA testing, or outside of the cancer screening context). Although it is true that some fears, such as of partner abandonment, were only expressed by women, this does not necessarily mean that they are not experienced by men—only that we did not find them in the sample of studies included in this review. This gender bias may just be an artefact of the types of screening included in the review, but future studies should address the validity of this taxonomy of cancer fears in men. In addition, some fears may be more prevalent for certain types of cancer. For example, womanhood issues may be more prevalent for breast cancer than for colorectal cancer, and fears of being blamed or stigmatised may be more prevalent for lung cancer than for some other types of cancer. The aim of this review, however, was to explore and categorise the fears that cancer (in general) evokes in the general population, and not to explore differences in cancer fears between cancer types. Future studies should explore the prevalence of the various fears for different types of cancer. We relied on the published quotes and authors' interpretations for our analyses, which could mean we missed some fears that were not mentioned in the published manuscripts. The prevalence or population distribution of cancer fears could not be deduced from these qualitative studies.

Despite these limitations, we feel confident that we have presented a robust taxonomy of cancer fears in the context of cancer screening, because of the large number of triangulated studies, the diversity of the study samples, and the fact that the updated literature search only identified a single new subtheme. In addition, many of the fears described in this review have also been described in studies of other types of cancer screening,^58^, ^59^ help‐seeking for cancer symptoms^60^, ^61^ genetic cancer risk,^62^ and cancer patients,^63^ lending further support to the idea that the cancer fears identified in this review are universal.

## 11 CONCLUSIONS

This systematic review and synthesis of qualitative evidence drew out the multidimensionality of cancer fear. Cancer fears emanated from a core view of cancer as an enemy, evoking fears about its proximity, the (lack of) strategies available to keep it at a distance, the personal and social implications of succumbing, and dying from cancer. The view of cancer as an enemy seems widely reinforced in society but may impede effective cancer control strategies and the adoption of preventive health behaviours. Future policies should focus on removing mixed messages in the public portrayal of cancer.

## AUTHORS' CONTRIBUTIONS

CV, CvW, JWal, and JWar conceived the study. CV, MH, LM, and GB assisted CvW, JWal, and JWar in the design of the study, and conducted the systematic review, data extraction, and meta‐synthesis. CV and GB took the lead in writing the manuscript, with input from all other authors. All authors have seen and approved the final version of the manuscript before submission.

## DECLARATION OF INTERESTS

The authors declare that they have no competing interests.

*Included in meta‐synthesis

## Supporting information

Supporting info item

Supporting info item
