# Supplementary material for: What do people fear about cancer? A systematic review and meta‐synthesis of cancer fears in the general population
Source: Psychooncology. 2016 Oct 6;26(8):1070–9. doi: 10.1002/pon.4287 (PMC5573953; doi:10.1002/pon.4287)
Supplement: Supplementary file 1 — Supporting info item [file PON-26-1070-s001.pdf]

ONLINE SUPPLEMENT 1

Characteristics of included studies

No.	Author, year	Type of screening	Country	Aims of study	Participants	Data collection method	Reported data analysis method
1	Adejimi and Akinleye 2014	Cervical	Nigeria	Assess knowledge, attitude and practices of cervical cancer prevention of women attending an HIV treatment centre	38 HIV positive women	Focus groups	Thematic analysis
2	Agurto et al. 2004	Cervical	Venezuela Ecuador, Mexico, El Salvador, Peru	Identify cultural and socioeconomic frameworks that might negatively or positively affect women's behaviour in seeking preventive health services in Venezuela, Ecuador, Mexico, Peru, and El Salvador.	19 focus groups and 46 interviews with women	Focus groups and personal interviews	Various methods
3	Austin et al. 2009	CRC (FS)	UK	Explore perceived barriers to CRC screening among UK ethnic minority populations, and examine lay recommendations to increase participation	53 White British and ethnic minority men and women	Focus groups	Framework analysis
4	Azaiza and Cohen 2008	Breast and cervical	Israel	Explore the ways in which cultural beliefs and attitudes, religious precepts about breast cancer and early detection screening, and modern biomedical perceptions interact and shape the perceived barriers to and motivations for screening	51 Arab women	Focus groups	Content analysis
5	Bass et al. 2011	CRC (COL, FOBT)	US	Elucidate if/how gender and screening status may be related to perceptions of barriers to CRC screening in an African American primary care clinic	23 African American women and men	Focus groups	Not specified but analytic process described
6	Bastani et al. 2001	CRC (COL, FOBT, FS)	US	Understand barriers to screening and develop interventions to increase early detection practices among ethnically and socioeconomically diverse groups.	56 ethnic minority men and women	Focus groups	Context analysis
7	Beeker et al. 2000	CRC (COL, FOBT, FS)	US	Identify the range of attitudes, beliefs, and behaviours in older adults amenable to change through public education and targeted interventions.	White and African American men and women	Focus groups	Not specified but analytic process described
8	Bener et al. 2002	Breast	UAE	Explore the determinants of breast cancer screening behaviour and assist in the development of the [breast screening] program.	41 women	Focus groups	Not specified but analytic process described
9	Best et al. 2015	Breast	US	Engage a group of African American women to identify important spiritual elements to be included in health communication materials, develop a spiritually framed breast cancer screening message in response to their feedback	35 African American women	Focus groups and personal interviews	Not specified but analytic process described

10	Borrayo et al. 2005	Breast	US	Uncover the cultural explanatory models that medically underserved Latinas appear to use as a heuristic in their decision to engage in breast cancer screening.	58 South American, Cuban, Salvadorian, Puerto Rican and Mexican women	Focus groups	Grounded theory
11	Brouse et al. 2003	CRC (FOBT)	US	Identify and define barriers to colorectal cancer screening in low-income, underserved minority populations.	8 men and women	Personal interviews	Descriptive analysis
12	Buki et al. 2004	Breast	US	Explore in depth the perceived breast cancer screening barriers and facilitative conditions among immigrant women from Mexico, Puerto Rico, Cuba, El Salvador, and South America.	58 South American, Cuban, Salvadorian, Puerto Rican and Mexican women	Focus groups	Grounded theory
13	Busingye et al. 2012	Cervical	Uganda	Assess the acceptability of cervical screening using visual inspection and to determine the reasons why women might refuse to be screened by this method.	24-32 women	Focus groups	Thematic analysis
14	Cadman et al. 2012	Cervical	UK	Explore self-reported cervical screening history among women who have been sexually abused, explore barriers to attendance for cervical screening in this population and identify measures to improve the experience of screening for these women.	4 women (focus group)	Online survey with open-ended questions and online focus group	Content analysis
15	Cadman et al. 2014	Cervical	UK	Explore attitudes, views and understanding of women attending a Hindu temple in London, UK towards cervical screening, human papillomavirus (HPV) testing and two HPV self-sample collection devices.	23 Hindu women (focus groups)	Focus groups and survey	Framework analysis
16	Chapple et al. 2008	CRC (FOBT)	UK	Explore people's experience of screening and to understand decision-making to take part in screening or not.	44 White British and Black Caribbean men and women	Personal interviews	Thematic analysis
17	Cohen 2009	Breast	US	Examine how African American women understand the many uncertainties fundamental to cancer risk communication, particularly in the breast cancer context.	49 African American women	Focus groups	Not specified but analytic process described
18	Denizard-Thompson et al. 2014	CRC	US	Examine the utility of text messages to support colorectal cancer screening.	27 men and women	Focus groups	Not described
19	Dolezil et al. 2014	CRC, breast, and prostate	Germany	Explore motives and attitudes towards cancer screening among the elderly.	64 men and 56 women	Personal interviews	Grounded theory
20	Duran 2011	Cervical	Turkey	Examine woman's attitudes and thoughts towards cervical cancer and its early diagnosis using the Health Belief Model.	11 women	Personal interviews	Content analysis and thematic coding

21	Ealey et al. 2011	CRC<br>(FIT and COL)	US	Report qualitative findings from focus groups with patients in [primary care centres] to inform the development of a low literacy DVD and photonovella booklet to prepare patients for provider discussions about CRCS, particularly I-FOBT use.	39 ethnically diverse men and women	Focus groups	Thematic coding
22	Ekberg et al. 2014	CRC<br>(FOBT)	UK	Investigate the attitudes of individuals living in the East Midlands of England towards the NHS bowel cancer screening programme.	33 men and women	Focus groups	Not described
23	Engelman et al. 2012	Breast	US	Determine the unique mammography-related experiences that women of two racial/ethnic groups perceive to be of primary importance.	88 Hispanic and non-Hispanic white women	Focus groups	Ethnography with content analysis
24	Ersin and Bahar 2013	Cervical	Turkey	Investigate perceived inhibiting and facilitating factors concerning cervical cancer early diagnosis behaviour in Turkish women over the age of 40	35 women	Focus groups	Content analysis
25	Fang and Baker 2013	Cervical	US	Understand the barriers and facilitators of cancer screening among Hmong women.	44 Hmong women	Focus groups	Not specified but analytic process described
26	Fernandez et al. 2005	Breast	US	Identify factors influencing repeat mammography screening among low-income African American and Hispanic women.	58 African American and Hispanic women	Focus groups and personal interviews	Grounded theory
27	Filippi et al. 2013	CRC<br>(COL, FOBT, FS)	US	Explore perceptions of American Indian women older than 50 years toward CRC screenings, existing barriers, and suggestions to promote education and screening among American Indian women in the Midwest.	52 American Indian women	Focus groups	Not specified but analytic process described
28	Fowler 2006	Breast	US	Describe the social processes used by African American women ages ≥50 years in making decisions about mammography screening.	30 African American women aged 50 or over	Personal interviews	Grounded theory
29	Friedman et al. 2012	Breast and cervical	US	Provide further insight into the perspectives of women who are obese, to examine in greater depth their barriers to both cervical and breast cancer screenings, and to understand why some women who are obese undergo cancer screening whereas others do not.	33 Black and White obese women	Personal interviews	Grounded theory
30	Frisby 2002	Breast	US	Determine what effect attitudes and perceptions of breast cancer have on underutilization of and experience with mammograms, provide insights on how communicators might address underserved populations, and provide insights on creating effective breast cancer campaigns.	92 Black women	Survey with open-ended questions	Content analysis

31	Goldsmith and Chiaro 2008	CRC (COL, FOBT, FS)	US	Draw out details of patients' concerns regarding CRC screening and to solicit their thoughts on how physicians could address and even resolve these issues.	15 men and women	Focus groups	Not specified but analytic process described
32	Granado et al. 2014	Breast	Barbados	Explore experiences with mammography among Barbadian women by investigating how barriers are negotiated in a setting of resource-constrained health care provision without a national screening programme.	110 women	Focus groups	Content analysis
33	Greaney et al. 2014	Breast, cervical, CRC	US	Explore perceptions of and barriers to cancer screening, understand audience communication norms related to design of interactive voice response messages	40 Latina women	Focus groups	Thematic content analysis
34	Greco et al. 2010	Breast	US	Describe and explain how women 55 years of age and older with at least one first-degree relative diagnosed with breast cancer make screening mammography decisions.	23 women	Personal interviews	Brief grounded theory
35	Green et al. 2008	CRC (COL)	US	Identify potential barriers to screening colonoscopy among low income Latino and white non-Latino patients in an urban community health centre.	40 Latino and white non-Latino men and women	Personal interviews	Content analysis
36	Greiner et al. 2005	CRC (COL, FOBT, FS)	US	Explore colorectal cancer screening knowledge, attitudes, barriers, and preferences among urban African Americans.	55 African American men and women	Focus groups	Not specified but analytic process described
37	Greisinger et al. 2006	CRC (COL, FOBT, FS)	US	Determine the current level of awareness and understanding about CRC and CRC screening.	42 ethnically diverse men and women	Focus groups	Not specified but analytic process described
38	Griffiths et al. 2010	Breast	UK	Examine how the nature of medical technology influences women's negotiated positions in relation to medicalization; to explore women's accounts of breast screening, comparing accounts of mammography screening, and breast awareness undertaken by women themselves.	61 women	Personal interviews	Not specified but analytic process described
39	Guilfoyle et al. 2007	Cervical	US	Investigate how low-income, African American and Hispanic older women make decisions about cervical cancer screening and to describe the supports and barriers to their screening practices.	98 African American and Hispanic older women	Focus groups	Content analysis
40	Hennelly et al. 2015	CRC	US	Describe key beliefs that exist about screening colonoscopy, generate a theory of colonoscopy decision-making using a category approach, and identify possible targets within the decision-making process for stories to be used to encourage a reader to complete a screening colonoscopy.	12 Latino men and Latina women	Personal interviews	Not specified but analytic process described

41	Holmes-Rovner et al. 2002	CRC (FOBT)	US	Investigate factors that may encourage those who know about screening but do not follow through to participate in screening.	21 African American and White men and women	Focus groups	Not specified but analytic process described
42	Ishida et al. 2001	Breast	US	Explore health beliefs and attitudes of Samoan women toward early detection of breast cancer and use of mammography.	15 Samoan women	Personal interviews	Content analysis
43	Jackson et al. 2000	Cervical	US	Obtain information about the cervical cancer screening behaviour of Cambodian Americans.	42 Cambodian American women	Personal interviews	Ethnography
44	Jepson et al. 2007	Breast, cervical, CRC	UK	Identify what information people want in order to feel informed when making a choice over whether or not to be screened; to identify what information people use to make the choice about screening; to gain an understanding of the relationships between information and knowledge, choice and behaviour; to compare the differing types and amount of information that people want for each screening test (breast, cervical and colorectal)	68 men and women	Focus groups and personal interviews	Grounded theory
45	Jilcott Pitts et al. 2013	CRC (COL, FOBT, FS)	US	Examine barriers and facilitators to CRC screening among low-income, rural, minority eastern North Carolina residents.	45 White and Black, non-Hispanic men and women	Focus groups	Not specified but analytic process described
46	Jones et al. 2010	CRC (BE, COL, FOBT, FS)	US	Understand current perspectives on CRC screening by primary care patients.	40 African American and White men and women	Focus groups and survey with open-ended questions	Not specified but analytic process described
47	Kahn et al. 1999	Cervical	US	Explore adolescent girls' understanding and perceptions of Pap smears and barriers to compliance.	27 ethnically diverse adolescent girls	Focus groups and personal interviews	Content analysis
48	Kahn et al. 2005	Breast	US	Uncover attitudes and perceived barriers to mammography among women diagnosed with mental illness and/or substance use disorders; to obtain women's explanations for why they do/do not adhere to mammography screening recommendations.	26 ethnically diverse women with mental illness/substance abuse disorders	Focus groups	Brief grounded theory
49	Kaltsa et al. 2013	Breast	Greece	Examine women's perceptions of mammography screening, understand the factors that influence screening behaviour.	33 women	Personal interviews	Not described
50	Kawar 2013	Breast	US	Explore the perceived barriers related to breast cancer screening among US Jordanian Palestinian immigrant women.	Jordanian and Palestinian immigrant women	Personal interviews	Structured content analysis

51	Kelly et al. 2015	Cervical	US	Obtain a more complete and theoretically informed understanding of the role of cancer worry in cervical cancer screening among Appalachian women.	24 women	Personal interviews and quantitative survey	Thematic coding
52	Keshevarz 2012	Breast and cervical	Iran	Understand the effective factors on the breast and cervical cancer screening behaviour.	70 women	Focus groups	Content analysis
53	Khazaee-pool et al. 2014	Breast	Iran	Explore perceptions of breast cancer screening among Iranian women who have never had a mammogram.	16 women	Personal interviews	Content analysis
54	Kissal and Beşer 2011	Breast	Turkey	Investigate experiences of breast self-examination, clinical breast examination and undergoing mammography, and perceived barriers among elderly women aged 60-75 years.	46 elderly women	Focus groups	Content analysis
55	Kwok et al. 2005	Breast	Australia	Explore and investigate the factors associated with mammographic decisions of Chinese-Australian women.	20 Chinese-Australian women	Personal interviews	Thematic analysis
56	Kwok et al. 2006	Breast	Australia	Examine the role of culture in breast health practices and explanations of breast cancer and its association with risk perception among Chinese–Australian women.	20 Chinese-Australian women	Personal interviews	Thematic analysis
57	Lee 2000	Cervical	US	Determine both the amount and type of knowledge Korean-American women have about cervical cancer, the barriers to cervical cancer screening, and the motivators that facilitate early screening.	102 Korean-American women	Focus groups	Content analysis
58	Lee et al. 2014	Breast, cervical, and stomach	Republic of Korea	Assess the barriers to cancer screening in people with low socioeconomic status	23 men and women	Focus groups	Directed content analysis
59	Logan and McIlfatrick 2011	Cervical	UK	Explore the experiences and perceptions of cervical screening among women from a socially deprived area in a region in the UK.	48 women	Focus groups	Thematic content analysis
60	Lyttle and Stadelman 2006	Breast and cervical	US	Obtain an understanding of attitudes and opinions about breast and cervical cancer screening tests; to determine motivational factors that would encourage low-income women to be screened for breast and cervical cancer; and to evaluate selected breast and cervical cancer educational materials according to perceived effectiveness by women.	69 women	Focus groups	Not described
61	Marlow et al. 2015	Cervical	UK	Explore self-perceived barriers to cervical screening attendance among ethnic minority women compared to white British women.	43 ethnic minority women and 11 White British women	Personal interviews	Framework analysis
62	Meana et al. 2001	Breast and cervical	Canada	Identify meanings of breast cancer and breast cancer screening held by older immigrant Tamil women.	30 older immigrant Tamil women	Focus groups	Content analysis

63	Molina-Barcelo et al. 2011	CRC (FOBT)	Spain	Examine the factors influencing participation and non-participation in a CRC screening programme, analysing the differences by gender and SES to gain a deeper understanding of the social determinants of health.	56 men and women	Focus groups	Discursive analysis
64	Naish et al. 1994	Cervical	UK	Determine the factors which deter non-English speaking women from attending their general practitioner for cervical screening in the city and east London area.	66-110 ethnically diverse women	Focus groups	Not described
65	Ndikom and Ofi 2012	Cervical	Nigeria	Explore the factors influencing utilization of cervical screening services among women in selected health facilities in [Nigeria].	82 women	Focus groups	Not specified but analytic process described
66	Nekhlyudov et al. 2003	Breast	US	Explore the complex aspects of the decision-making process to learn which factors are involved in women's decisions about screening mammography, what role medical providers play in these decisions, and what women's preferences are for information regarding screening and involvement in making these decisions.	16 White and African American women	Personal interviews	Not specified but analytic process described
67	Nolan et al. 2015	Cervical	US	Explore factors that might lead to delays in appropriate cervical cancer screening and diagnosis among Black women in Massachusetts.	64 Black, non-Hispanic women	Focus groups	Not specified but analytic process described
68	O'Brien et al. 2009	Cervical	Canada	Gain insights into attitudes toward cervical cancer screening and beliefs about cervical cancer among First Nation Cree women.	8 First Nation Cree women	Personal interviews and observation	Focused ethnography
69	Ogedegbe et al. 2005	Breast, cervical, CRC (FOBT, FS)	US	Explore perspectives of patients from various low-income and minority backgrounds with regards to multiple cancer screening behaviours.	187 African American and Latina women	Personal interviews	Content analysis
70	Opoku et al. 2012	Breast	Ghana	Determine population-based rates of reported breast cancer screening; to assess breast cancer-related knowledge, attitudes, beliefs among Ghanaian women, and to explore their relation to screening practices.	10 breast cancer patients, 10 breast clinic attendees, 3 consultants, 2 traditional healers	Personal interviews and quantitative survey	Not described
71	Oscarsson et al. 2008	Cervical	Sweden	Describe and interpret why women with no cervical smear taken during the previous 5 years choose not to attend a cervical cancer screening programme.	14 women	Personal interviews	Inductive content analysis
72	Paz-Soldán et al. 2010-2011	Cervical	Peru	Explore women's knowledge concerning cervical cancer and Pap smears, identify their perceived barriers to obtaining Pap smears, and describe possible intervention ideas.	177 women	Focus groups	Not specified but analytic process described
73	Peek et al. 2008	Breast	US	Explore the underlying reasons for the fear and fatalism associated with breast cancer screening among low income African-American women in Chicago.	29 African American women	Focus groups	Content analysis

74	Percac-Lima et al. 2013	Breast	US	Assess Bosnian, Iraqi, and Somali refugee women's perspectives on preventive care and perceived barriers to breast cancer screening.	57 women	Personal interviews	Content and thematic analysis
75	Pietrzak et al. 2011	Breast	Poland	Assess breast cancer screening programme attitudes.	Women	Focus groups	Not described
76	Purtzer and Overstreet 2014	Breast	US	Investigate the responses to experiences that perpetuate non-screening or facilitate screening within the context of transformative learning theory.	25 women	Focus groups	Not specified but analytic process described
77	Remennick 2003	Breast	Israel	Shed more light on the sociocultural determinants of immigrant women's early detection of breast cancer and factors that impede participation in breast cancer preventive programmes.	23 Russian immigrant women	Focus groups and personal interviews	Not described
78	Ritvo et al. 2013	CRC (COL, FOBT, FS)	Canada	Pinpoint gender-specific obstructive attitudes to CRC screening.	81 men and women	Personal interviews	Not specified by analytic process described
79	Royak-Schaler et al. 2004	Breast and CRC	US	Investigate perceptions of personal cancer risk, understanding of risk factors and risk reduction behaviour, and strategies for delivering risk information to low-income African-American and Hispanic women.	42 African American and Hispanic women	Focus groups	Content analysis
80	Saadi et al. 2011	Breast	US	Assess perspectives on preventive care and perceived barriers to breast cancer screening among Iraqi refugees.	Female Iraqi refugees	Personal interviews	Content and thematic analysis
81	Salazar 1996	Breast	US	Identify the facilitators and barriers that influence the mammography utilization patterns of a group of low-income, rural, Hispanic women.	29 Hispanic women	Focus groups and personal interviews	Content analysis
82	Scarinci et al. 2013	Cervical	US	Examine the knowledge, beliefs, and attitudes regarding cervical cancer and HPV infection, as well as acceptability and usability of self-collected sampling for HPV testing	96 African American women	Focus groups	Not specified but analytic process described
83	Schoenberg et al. 2005	Cervical	US	Provide updated lay perceptions of factors that influence cervical cancer screening among Appalachian women.	25 middle-aged Appalachian White and Black women	Personal interviews	Content analysis
84	Sly et al. 2013	CRC (COL)	US	Understand why patients who received patient navigation services did not complete a colonoscopy.	16 African American men and women	Personal interviews	Thematic content analysis
85	Suh 2006	Breast	US	Present cultural meanings of breast, breast cancer, and breast cancer screenings in Korean immigrant women.	15 Korean immigrant women	Focus groups	Thematic analysis
86	Taha et al. 2012	Breast	Jordan	Explore Jordanian women's views and perceptions about breast cancer and breast health.	64 women	Focus groups	Latent content analysis

87	Tejeda et al. 2009	Breast	US	Describe barriers to and facilitators of breast cancer screening and how people in a woman's social network influence these screening behaviours.	40 Mexican, Mexican-American, or Hispanic women	Personal interviews	Not specified but analytic process described
88	Teng et al. 2014	Cervical	Uganda	Develop an understanding of the role of embarrassment in relation to cervical cancer screening and self-collected human papillomavirus DNA testing	6 key informant health workers and 16 local women	Focus groups and personal interviews	Thematic analysis
89	Tessaro et al. 2006	CRC (BE, COL, FOBT, FS)	US	Examine knowledge of and barriers to colorectal cancer screening and predictors of adherence to screening guidelines in an Appalachian church population aged 50 years and older.	205 White Appalachian men and women	Focus groups	Not specified but analytic process described
90	Tkatch et al. 2014	Cancer screening	US	Investigate the barriers to cancer screening among Orthodox Jewish women in the metropolitan Detroit area.	18 Orthodox Jewish women	Focus groups	Content analysis
91	Todd and Stuifbergen 2011	Breast	US	Gain an understanding of barriers and facilitators to mammography and breast self-examination experienced by women with multiple sclerosis (MS).	36 women with MS	Personal interviews	Content analysis
92	Trigoni et al. 2008	Breast	Greece	Explore the knowledge, attitudes and perceived practices of both primary care physicians and women in relation to mammography breast screening on the island of Crete in Greece.	30 women	Personal interviews	Thematic analysis
93	Truesdale-Kennedy et al. 2011	Breast	UK	Describe understanding of breast cancer and experiences of breast mammography among women with an intellectual disability.	19 women with an intellectual disability	Focus groups	Thematic content analysis
94	Varela et al. 2010	CRC (COL)	US	Gain a better understanding of the factors (both barriers and facilitators) that affect the decision, by Hispanics, to have (or not have) screening colonoscopies.	35 Hispanic men and women	Focus groups	Not described
95	Wackerbarth et al. 2005	CRC (BE, COL, FOBT, FS)	US	Identify perceptions of both the barriers and benefits to colorectal screening.	30 men and women	Personal interviews	Not specified but analytic process described
96	Willis and Baxter 2003	Breast	Australia	Determine what health beliefs and social supports may contribute to decision making around screening for breast cancer for women who have not been invited to screening.	14 women	Personal interviews	Not specified but analytic process described
97	Wittenberg et al. 2015	Cervical	US	Develop a method to measure homeless women's decision-relevant preferences with respect to cervical cancer screening interventions	42 homeless women	Focus groups and quantitative survey	Thematic analysis
98	Wong et al. 2008	Cervical	Malaysia	Explore sociocultural issues relevant to cervical cancer screening practices among Malaysian women who have never had a Pap smear.	20 women of ethnically diverse backgrounds	Personal interviews	Not described

99	Wood et al. 1997	Cervical	South Africa	Ascertain rural Black women's constructions and understandings of cervical symptomatology and screening.	55 Black women from 3 different regions	Personal interviews	Not described
100	Wood and Della-Monica 2006	Breast	US	Report on participant evaluation of the desirability, usability and relevance of a video kit to increase knowledge about breast cancer and screening practices among older Caucasian and African-American women, as well as to assess personal experiences with the screening procedures of mammography and BSE, both prior to and following the intervention.	23 African American, Caucasian and Hispanic women	Focus groups	Not specified but analytic process described
101	Wood and Della-Monica 2011	Breast	US	Investigate the perceptions of older Black and White women surrounding their personal understanding of breast cancer risk.	36 Black and White elderly women	Focus groups	Content analysis
102	Woodrow et al. 2008	CRC (FOBT)	UK	Explore the perceptions with regard to the communication of information designed to promote informed choice in the bowel cancer screening programme.	86 men and women	Focus groups	Not specified but analytic process described

Abbreviations: BE = Barium enema, COL = colonoscopy, CRC = colorectal cancer, FOBT = faecal occult blood test, FIT = faecal immunochemical test, FS = flexible sigmoidoscopy, HPV = human papilloma virus, MS = multiple sclerosis, UAE = United Arab Emirates , UK = United Kingdom, US = United States of America
