# Supplementary material for: What do people fear about cancer? A systematic review and meta‐synthesis of cancer fears in the general population
Source: Psychooncology. 2016 Oct 6;26(8):1070–9. doi: 10.1002/pon.4287 (PMC5573953; doi:10.1002/pon.4287)
Supplement: Supplementary file 2 — Supporting info item [file PON-26-1070-s002.pdf]

ONLINE SUPPLEMENT 2

What do people fear about cancer? A systematic review and meta-synthesis of cancer fears in the general population- Detailed results and discussion

This document is the online supplement to the manuscript by Vrinten C, McGregor LM, Heinrich M, von Wagner C, Waller J, Wardle J, & Black GB: 'What do people fear about cancer? A systematic review and meta-synthesis of cancer fears in the general population'. This supplement provides a more detailed description of the results of the meta-synthesis, including supporting quotations, and puts these into the context of other research findings.

Meta-synthesis

We found that people's fears were related to an overarching meaning of cancer itself as something that is almost universally dreadful, disfiguring, and incurable, that causes tremendous emotional and physical suffering, returns endlessly, and ultimately kills you:[1-6]

> [Participants indicated] that they thought cancer was a terrible, painful disease that caused suffering and death to many. From the participants' perspective, this was the meaning of cancer. (Ishida et al., 2001, breast, US)[1]
>
> *"[Cancer] means body disfigurement, suffering, family life disruption, and death."* (Taha et al., 2012, breast, Jordan)[6]

We have labelled this view of cancer as 'cancer as the enemy' (first theme). In our interpretation, the perception of this enemy is at the root of people's cancer fears, and provokes the following threat appraisals:

- How close am I to this enemy? (second theme)
- How do I keep the enemy at bay? (third theme)
- What if the enemy attacks? (fourth theme)
- And finally, what if the enemy wins? (fifth theme)

People's fears about cancer did not seem to follow a linear pathway along the consecutive appraisal stages. Rather, fears from different appraisal "stages" could be experienced simultaneously.

1. Cancer as the enemy

The first theme explores the root of people's cancer fears. Participants in approximately a quarter of all included studies talked about cancer not just as a disease, but as a sentient and malicious persona with negative personality traits, such as viciousness, unpredictability, and indestructibility:[6-12] *"I think also the fear with cancer is that it seems to come out of nowhere."*[13] People described cancer as lurking inside your body, spreading itself stealthily and inescapably,[3, 8, 9, 11, 12, 14] and waiting to be discovered.[14] By the time it revealed itself, many believed it would be too large and strong to do anything,[3, 10] except to "*figure out how to fix the next person*",[10] which created a sense of betrayal:

> *"The cancer is a traitor… You can be examined all the time… and nothing comes up, and then when you find out you have cancer it's too late."* (Guilfoyle et al., 2007, cervical, US)[11]
>
> *"Yeah, that's what is so urgent about cancer. You may not even know you have it and all of a sudden, you go to the doctor and they say, "You've had this for years," or whatever. It's like it lays dormant in*

> *your body […]. And it's almost impossible not to have cancer in the back of all of our minds because many times you're conscious of your body and you look in the mirror at yourself, you know that you can be a potential cancer risk. It can happen."* (Cohen, 2009, breast, US)[14]

Echoing its strong association with death (see theme 5), cancer was seen as a killer[6, 8, 14-18] that comes out of nowhere[13] and will always return[6, 11, 13, 19] despite treatment:

> *"I always heard from the stories that once it's in you, it just goes straight through you."* (Fowler, 2006, breast, US)[10]

> *"If you find it early, you can get treated for it, but I think that once the cancer is there, it's always gonna come back. I don't care how many years you go, that cancer's always gonna come right back."* (gender unknown, Greiner et al., 2005, CRC, US)[19]

This description of cancer's personality resembles its portrayal by the mass media, as insidious, random, secretive, stealthful, and almost inevitable.[20]

Participants in several studies described cancer as parasitising organisms that are alien to the body and infect[19] the body like viruses,[6] bacteria,[6] or worms:[4, 7, 11, 21] *"[…] they say the cancer is, starts in one side and then it spreads and spreads, like a little worm that goes through all the organs."*[21] Some people believed that cancer was infectious or could be transmitted to others:[4, 6, 7, 12, 19, 22, 23] *"The husband said that this is a virus, a small organism inside the body which eats from the body. It would be possible that it can be transferred to him and live upon him too."*[6] In some studies, cancer was characterised by botanical properties, such as taking root and making you rot.[4, 14, 16] Notions of cancerous growth were strongly related to destructive qualities, such as burning, rotting, or being eaten away:[4, 6, 21, 24, 25] *"[the cancer] started in her breast, and ate everything, the entire breast . . . it got to the heart and she died…"*[24]

The aggressive personality and invasive properties of cancer were a source of much anxiety and fear:[6, 8, 11, 12, 14, 16, 24] *"Cancer is a scary thing, period. By the time it's detected it spreads all through your body. It's too late."*[10]

2. How close am I to the enemy?

In more than half of included studies, participants assessed their own relationship to cancer in terms of proximity. Generally, greater proximity to cancer increased fear of cancer, and a greater distance reduced fear and created a feeling of safety. Proximity was informed by cancer encounters, the presence of symptoms, and assessments of personal risk.

Cancer encounters.

Past encounters with cancer created a series of expectations which could provoke fear. Encounters with cancer could be indirect, such as stories related by one's social connections or the mass media.[5, 6, 9, 11, 12, 15, 26-30] Although mass media and outreach campaigns could potentially allay fears about cancer and encourage early detection behaviours,[6] some authors[11, 15, 29] observed that they *"led to confusion and had created or enhanced fears about cancer and (cancer) screening"*:[29]

> *"There was a cancer campaign advertisement selling t-shirts with a specific symbol on them, which made me feel a lot of fear about breast cancer and made me even stop thinking of taking part in its early detection. The way the advertisement was presented, gave me the impression that one day all women will be threatened. So, it made me even change the television channel in order not to hear about it at all."* (Kaltsa et al., 2013, breast, Greece)[29]

Despite the influence of the media, cancer fears were mainly shaped by direct encounters with cancer, such as witnessing the effects of cancer in friends and family.[6, 8, 9, 11, 13-16, 24, 25, 29-41] In about a

quarter of included studies, participants related their fears to having witnessed these consequences in others. Personal experiences ranged from seeing others suffer the effects of chemotherapy and surgery,[4, 8, 25, 32, 34, 38, 39] to knowing people who had died of cancer.[6, 8, 11, 13, 14, 16, 29-33, 35-37] Cancer encounters inspired fear through contemplation of one's own fate:

> *"My son's stepmother … was diagnosed with bilateral breast cancer. It brought up a lot of fear for myself because the first thing I thought about (was) 'My God, it spread to the lymph nodes and it's pretty serious…' […] I don't know if I could handle that."* (Purtzer and Overstreet, 2014, breast, US)[39]

> Women […] who reported having had a previous mammogram also used the word fear to describe what encouraged them to have a mammogram […]: *"I don't like being without [a mammogram] for a long time. So many people have died of cancer. I'm afraid. A friend died of cancer. I was with her [when she died], oh my God, it was such sadness."* (Tejeda et al., 2009, breast, US)[31]

Some respondents also reported personal 'cancer scares', such as the discovery of a (benign) breast lump:[6, 35, 39] *"I definitely did have a mass in my left breast […] Fortunately for me, it was benign… When I go for the mammogram, I'm still nervous because of that incident."*[35]

For most people, the enhanced fear of cancer brought about by cancer encounters seemed to motivate them to attend screening:[6, 9, 25, 29-33, 39, 41]

> *"I don't like being without (a mammogram) for a long time. So many people have died of cancer. I'm afraid."* (Tejeda et al., 2009, breast, US)[31]

> Women felt fear of developing breast cancer especially after experiencing a friend or relative's death from the disease. This led the majority of them to act against a possible scenario of identifying breast cancer at a late stage by having regular breast screening. […]: *"I was shocked when a friend of mine died from the disease. That incident made me realise […] how dangerous (breast cancer) can be if not detected at an early stage. So, after that I said to myself that 'either you like it or not, you are now going to have mammograms on a regular basis'. […]"* (Kaltsa et al., 2013, breast, Greece)[29]

For some participants, however, the fear inspired by cancer encounters promoted screening avoidance, usually because of coexisting beliefs that cancer is uniformly fatal:[15, 29, 30, 36, 38, 42]

> *"(…) My sister's breast cancer was a terrible one, which finally destroyed her whole chest. So that is why I don't go for breast screening, […], I am afraid."* (Kaltsa et al., 2013, breast, Greece)[29]

> All informants had some first-hand experience with cancer in their close ones and knew how harsh (and often futile) cancer therapy may be. If treatment means prolonged suffering without guaranteed cure, many women opted for *"I don't want to know it before time"* mind-set. […] The combination of fear and disbelief in current cancer treatment provided a fertile ground for a fatalist outlook that was often translated in passive or negative attitudes towards early detection practices. (Remennick, 2003, breast, Israel)[15]

The importance of personal experience – rather than media – in shaping people's fears suggests that the scope for social marketing techniques to be used to change cancer's image may be limited.

Symptoms.

Cancer fear was related to cancer symptoms in various ways. Cancer's potential to be asymptomatic in its early stages was a source of anxiety, but only in a very small minority of studies:[9, 24, 31, 36]

> *"(…) I wanted to do that [mammogram] because of fear, because they say that everything can be fine, nothing hurts, and sometimes you come out with it [breast cancer]."* (Tejeda et al., 2009, breast, US)[31]

Instead, about a quarter of all included studies mentioned that people felt reassured by an absence of symptoms, which was seen as an indication that there was no need to worry about cancer or attend cancer screening.[1, 2, 5-7, 12, 18, 19, 27, 32, 33, 37, 39, 41, 43-54] *"I feel healthy, and I don't feel like I have to worry"*[48] was a frequently given reason for not attending screening. A dominant belief was that medical services are only for the sick,[2, 27, 33, 43] and that "*if one looks and feels okay, one must be okay"*:[19]

> *"I think I don't need to do it [the test] now because, thank God, at this moment I have no symptoms."* (woman, Molina-Barcelo et al., 2011, CRC, Spain)[50]

For many, only the appearance of symptoms would signal advancing cancer and prompt them to seek medical help or attend screening.[2, 5-7, 12, 16, 18, 27, 33, 39, 46] Pain, in particular, was seen as an important cue to action:[7, 12, 16, 33, 46] *"If I'm not in pain, I think there's probably no need [to go for screening]."*[7]

Relying on symptoms to gauge the need to attend screening was discussed in about a quarter of studies, which echoes quantitative studies that have found that an absence of symptoms is endorsed as a barrier to screening by a quarter to a third of participants.[55, 56] The reliance on symptomatic cues for screening by those who are not worried about cancer contrasts with the view of cancer as a dangerous and unpredictable enemy, but lends further support to our interpretation that cancer fears emanate from this idea of cancer as an enemy. From a public health perspective, using symptoms as a compass of when to attend screening is a target for behaviour change, because the purpose of cancer screening is to detect *pre-symptomat*ic abnormalities in order to improve treatment success and survival.

Assessment of risk.

Perceptions of causation, control and risk of cancer were interrelated factors indicating proximity to cancer. People distinguished between causes and risks that were perceived to be under individual control from those that were not. Only fears associated with uncontrollable risks are discussed here; controllable risks are discussed in the next section as strategies to keep cancer at bay.

A number of uncontrollable cancer risks were mentioned, such as age and pollution, [7, 11, 12] but only familial or genetic factors, which were mentioned in relation to fear in 15 studies,[5, 6, 8, 14, 24, 27, 30, 32, 34, 36, 54, 57-60] strongly influenced people's cancer fear. Fears about familial risk of cancer ranged from mild concern and unease to confusion, sleeplessness, and psychological trauma.[5, 8, 14, 27, 34, 36] Because familial risk could not be influenced directly, several authors noted that participants tried to find other ways to allay their fears, such as by adjusting their diet,[14, 54] or through screening and early diagnosis:[5, 6, 27, 32, 34, 36, 54]

> *"I don't have breast cancer, but I do have cancer in my family, and so that is what I'm afraid of. That's one reason I have my Pap smear and my mammogram"* (Salazar, 1996, breast, US)[27]

> *"My uncle's daughter had cancer and her breast was removed. To be frank, I feel uneasy. I check my breasts. Women with a family member with breast cancer should have regular screening."* (Kissal and Beşer, 2011, breast, Turkey)[5]

> *"I had a sister that died from breast cancer and I know I'm next. It's very important to me to know that self-examinations can prevent cancer at an early stage […] and how important it is to get a mammogram at least once a year."* (Wood and Della-Monica, 2006, breast, US)[34]

For some participants, familial risk was such a strong risk indicator that a *negative* family history led them to believe that there was no need to worry about cancer or attend screening:[24, 57, 59, 60]

> I do not need to (attend cervical cancer screening): *"(…) There's no one in my family that has ever had any problems (with cervical cancer). (…) So there's no worries."* (Oscarsson et al., 2008, cervical, Sweden)[60]
>
> *"So many women believe that it's hereditary and that if they don't have it in the family, then what's the purpose of doing it [getting a mammogram]?"* (woman, Royak-Schaler et al., 2004, breast and CRC, US)[57]

The belief that screening is not necessary in the absence of a family history of cancer also forms a target for behaviour change, because only a minority of common cancers are associated with a hereditary predisposition.[61]

A dozen studies described people who were not afraid of cancer at all, because they did not think about it, did not feel susceptible to it, or had never experienced it in anyone close to them. This was linked to a lack of motivation to attend screening:[7, 9, 17, 18, 30, 33, 36, 60, 62-65]

> Some women did not feel concerned about cervical cancer and as a result had delayed screening attendance in the past. These women felt cancer would not affect them: (…) *"It just never seemed hugely important… I was never afraid that it could be something that could happen to me."* (Marlow et al., 2015, cervical, UK)[30]
>
> (Some) women said they did not tend to worry in general and therefore they did not worry a lot about cancer: *"If it happens then I'll deal with it, but I don't focus on it."* (Greco et al., 2010, breast, US)[36]

In a small number of studies,[24, 45, 66] participants mentioned ethnicity as a factor that could ameliorate cancer fear. Participants from ethnic minority backgrounds in these studies seemed to think of cancer as a *"Western disease"* or a *"White women's disease"* (i.e. breast cancer) that they did not need to worry about because they were less susceptible to it, making screening unnecessary:[24, 45, 66]

> The [women's] attitudes rejecting of screening were based on […] concerns about whether mammograms are as effective for Chinese women as they are for Western women. This is based on their belief that breast cancer is a 'Western disease' and that Caucasian women are prone to breast cancer as a result of having larger breasts. (Kwok et al., 2005, breast, Australia)[66]

A perceived lack of susceptibility has previously been identified as an important barrier to breast and cervical cancer screening in Hispanic women.[67] Research in breast cancer shows that its incidence is indeed lower for some ethnic minorities,[68] but other studies suggest that this is due to differences in breast cancer risk factors such as breastfeeding, and that incidence rates are similar across ethnic groups once these differences are controlled for.[69, 70] Some studies suggest that certain ethnic minority groups have a higher cancer burden,[71-73] which is partly due to their lower uptake of screening.[74, 75] Future research should address to what extent the belief that ethnic minorities are at lower risk of cancer underlies ethnic differences in cancer prevention behaviours.

3. How do I keep the enemy at bay?

Negative emotional states such as fear seldom remain unregulated.[76] This third theme explores fears and worries associated with four different strategies to keep the enemy at bay. Two strategies are active ('protecting yourself' and 'checking up on the enemy through screening'), while the other two are passive ('trusting someone to intervene on your behalf' and 'ignoring the enemy').

Protecting yourself against cancer.

About a third of included studies mentioned that cancer fear was regulated through control of certain risks. Some things were believed to increase cancer risk, and these became objects of fear themselves, and participants worried about how to avoid them. Other things were believed to protect against cancer, and participants described how exposure to them led to an absence of fear.

Relatively few studies mentioned lifestyle factors, such as diet and exercise, as a means of controlling risks and allaying fears:[8, 13, 14, 24, 26, 34, 54]

> Women expressed discomfort with their loved ones' cancer fears and avoidance, and encouraged their loved ones at risk to take preventive action (e.g. eating a healthier diet rich in vegetables). One participant recounted in response to her husband who said that cancer was *"just his family's death sentence,"* she would *"keep telling him it doesn't have to be"*. As the person responsible for cooking and buying groceries she tried to change the way he ate. (Cohen, 2009, breast, US)[14]

> *"I also feel like there's really nothing I can do about it. I mean, you read the studies that say that, you know, you eat healthy and you know, you have a better chance or whatever, but I don't know; I feel like it's so, so common and it's just kind of like a scare."* (woman, Tkatch et al., 2014, cancer screening in general, US)[13]

Instead, the focus was largely on factors that were deemed risky, such as worrying, thinking, or talking about cancer.[2, 30] They believed that worrying induced stress, and that stress would facilitate cancer development, and so they worried about being worried about cancer; a kind of meta-worry:[2, 24, 26, 35, 60]

> *"Just by worrying can we become sick. People who have cancer admitted that they were worrying too much. I always worry and that I might get cancer."*(Meana et al., 2001, breast and cervical, Canada)[2]

> *"Well, (…) I really don't think of it (breast cancer risk) because I don't think it's a good idea to worry. Worry can bring on a lot of anxiety, which can bring on a lot of stress, and stress could be a component in that area."* (Wood and Della-Monica, 2011, breast, US)[35]

A recent systematic review of the lay understanding of cancer by those without a cancer diagnosis also identified the belief that worry or anxiety could cause cancer.[77] This belief may hinder effective communication about cancer prevention. Participants in a number of studies also expressed fears that thinking or talking about cancer may bring it about or *"invite trouble"*:[13-15, 19, 24, 39, 51, 78, 79]

> Participants avoided saying the word cancer fearing that just uttering the word would result in getting the disease. (Kawar, 2013, breast, US)[78]

> Fear of verbalising was associated with the anticipation of a negative [screening] result […]: *"Verbalising, for me, is bringing on something that I don't want to deal with."* (Purtzer and Overstreet, 2014, breast, US)[39]

> [The participants described] a reluctance within the community to use the word cancer […]. Using the term cancer could "call" it to them. Therefore, the deep fear of the actual disease is a significant barrier to screening: *"I think the number one reason is the fear, which is, I think is across the board, but I, I also think that there is a concept of you don't invite trouble. You just, you don't."* (woman, Tkatch et al., 2014, general cancer screening, US)[13]

Of the 49 studies about breast cancer screening, more than a quarter mentioned the screening itself as a risky activity that was best avoided. Some women worried about the radiation exposure involved in mammographic screening:[5, 6, 9, 10, 15, 27, 38, 43, 66]

> *"I heard that X-rays can cause cancer. What if I go for it [mammogram] today and the X-ray causes cancer to develop in my body?"* (Kwok et al., 2005, breast, Australia)[66]

> Seven [of thirty] women mentioned that fear of the effect of exposure to radiation put them off [attending mammography screening]. (Trigoni et al., 2008, breast, Greece)[38]

It has recently been shown that 60% of women overestimate the radiation exposure involved in mammography screening,[80] which may contribute to this type of worry, and may put them off screening.

Compressing the breasts was another reason for concern. Some women believed that physical injuries to the breast could cause cancer, and they worried about the squeezing and pressing involved in mammography:[5, 9, 22, 32, 36, 38, 66, 81]

> A prominent factor that emerged was that some women in this study had fears that the procedure would cause them physical harm and actually result in them developing cancer […]: *"I heard that they have to press your breasts until they can fit into the machine. I think that must really hurt the breasts. What if I was alright before and then it hurts my breasts and I develop cancer later. Who knows?"* (Kwok et al., 2005, breast, Australia)[66]

> [Women] talked about the discomfort of having their breast "squeezed". Some said they had heard that this could cause breast cancer. (Fernandez et al., 2005, breast, US)[32]

> A few women believed that mammography was harmful to the breasts and caused cancer […]: *"The mammogram squeezes and smashes the breasts, which may even lead to cancer in some cases."* (Kissal and Beşer, 2011, breast, Turkey)[5]

In addition, some women were worried that compressing an existing tumour during mammography would cause it to burst and spread:[82] *"I heard that if you have cancer and you go and have a mammogram done, and the machine squeeze(s) you like that, it can burst and cancer will spread."*[82] This type of fear may be similar to fears that manipulating a tumour during surgery may cause cancer to spread (see theme 4: 'What if the enemy attacks?')

Women who held these beliefs viewed not attending mammography as protective of breast cancer. Although these fears were only reported in the context of screening for breast cancer, they may have implications for other types of cancer screening that involve radiation exposure (e.g. lung cancer screening), or manipulation of a potential tumour site (e.g. testicular cancer self-examinations). Beliefs that worrying or talking about cancer, stress, and physical trauma may cause cancer to develop could be a sign of the enigmatic and almost mythical origin of cancer to those who are afraid of cancer. Consistent with the idea that negative emotional states such as fear seldom remain unregulated,[76] they may need to be understood as attempts to gain some control over this deadly disease.

Participants also mentioned factors that they believed to be protective of cancer. Breastfeeding was mentioned in a number of studies about breast cancer screening.[5, 6, 12, 24, 83] Having breastfed was viewed as protecting against breast cancer,[6, 12, 24, 83] while not having breastfed was associated with increased breast cancer risk and increased fear of breast cancer:[5, 6]

> [About half of the informants] believed breast-feeding is a protective measure against breast cancer and they translated this into an elimination of personal risk. As one woman stated: *"I am not afraid of [breast cancer] because I have breast-fed all my children."* (Kwok et al., 2006, breast, Australia)[83]

Controllable risks were much less frequently mentioned in the context of cervical and colorectal cancer screening, although some studies mentioned that fear of cervical cancer was influenced by

having had children,[17, 52] and sexual activity.[11, 17, 27, 30, 45] Not being sexually active was seen as a reason for not having to worry about cervical cancer:[11, 30, 45]

> "*Because I'm not married I always used to think no I shouldn't be worried.*" (Marlow et al., 2015, cervical, UK)[30]

> [Women] often reported sexual inactivity as a reason why they did not have the Pap test: *"For me, that's when people marry, have children, you should go for a check-up. Because when you're young and if you don't have sex, you don't need it. I'm not worried because I don't have men."* (Guilfoyle et al., 2007, cervical, US)[11]

Check the enemy's progress through screening.

Screening presents an opportunity to obtain reassurance by checking up on cancer's progress, but about a third of included studies described how facing the prospect of this knowledge can (mostly temporarily) enhance cancer fear. Some participants had an intense fear that the test would reveal a cancer diagnosis, with some almost expecting it:[2, 22, 35, 36, 39, 46, 84-88]

> Women who seemed to relate the experience of screening with breast cancer diagnosis discussed their anxiety and fear associated with screening: *"When I get a mammogram, I hold my breath"* or *"I can't relax".* (Wood and Della-Monica, 2011, breast, US)[35]

> *"I kind of had trepidations to what they might find… you always worry every time."* (Purtzer and Overstreet, 2014, breast, US)[39]

> *"What if I get my sample? What results will I get? That fear of getting the results… Am I positive? Am I negative?"* (Teng et al., 2014, cervical, Uganda)[87]

> *"I was scared and wondering why they sent me for the test. I thought I had cancer and was thinking to inform my parents, brothers, and sisters that I won't live long."* (Meana et al., 2001, breast and cervical, Canada)[2]

> [W]omen described negative associations between prevention and disease, such that they were not just worrying about being diagnosed with breast cancer when they went for a mammogram, but were actually expecting it:
> *"Women might feel that every time you go for your mammogram, you got cancer."*
> *"[I'm afraid of] what they might find. That is my biggest fear. After that first [mammogram], I was a nervous wreck. I figured they would tell me I had breast cancer. […]"* (Peek et al., 2008, breast, US)[46]

A positive test result,[11, 15, 38, 78, 82, 84, 86, 89, 90] or in an extreme case merely the invitation to attend screening,[2] were often seen as a direct indicator of having cancer , which led to anxiety about the moment the results were received: *"The doctor told me… I was crying when I told him that my children are young, what did I have, what did the test show?"*[4] Many people worried about their reactions to a positive test result; expecting to cry, panic, be afraid, or be utterly in shock.[4, 15, 33-35] For some participants, fear of a positive test result was enhanced by earlier experiences of being recalled for further tests.[15, 35, 39]

> Previous experience with abnormal mammograms, particularly those requiring biopsies or additional testing, led to anxiety, worry, and fear: *"[…] When I go for the mammogram I'm still nervous because of that incident [i.e. an earlier false positive result]."* (Wood and Della-Monica, 2011, breast, US)[35]

This anxiety was sometimes enhanced by the delivery of results: having to wait *"a long time"* for test results provoked prolonged anxiety until the *"all clear"* was given:[14, 23, 36, 46, 84, 85, 88, 91-93]

> Respondents voiced […] their fear of having to wait for what was perceived as 'a long time' for results […]: *"I think it's really important how the results are delivered to you as well… getting the smear test itself isn't a problem… I didn't find it painful. All of that is okay. But for me it's the wait… It's getting the results, it's that waiting period."* (Logan and McIlfatrick, 2011, cervical, UK)[91]

> Because the women understood the purpose of the [cervical cancer screening] test, they were anxious until they knew their test result […]: *"[…] in the waiting period you don't know what to think. Until your test result (shows that) there's no abnormalities, then there's that sense of relief. […]"* (O'Brien et al., 2009, cervical, Canada)[92]

> A strong feeling of relief was also apparent once the women had received the results of the mammogram as they openly made aware to the group that they were 'clear' demonstrating their heightened anxiety levels leading up to the results. (Truesdale-Kennedy et al., 2011, breast, UK)[93]

Being told you would only be contacted if further tests or treatment were needed created prolonged anxiety about having missed the call:[23, 92]

> *"They said, "If there is a need for us to do surgery we'll contact you. If there isn't a need we won't call you." Like, you're just left hanging, waiting for the phone to ring or nervous because you're thinking you missed the call."* (O'Brien et al., 2009, cervical, Canada)[92]

Unclear communication of results, including benign findings, could also provoke anxiety:[10, 11, 24, 29, 46, 64, 82, 88]

> [One participant] received a letter […] informing her that calcium deposits were found on the mammogram. This letter provoked much anxiety. […]: *"[…] when I went, you know, the doctor says oh it's just calcium deposits, - which took 30 years off my life."* (Kahn et al., 2005, breast, US)[82]

For some people, the anxiety provoked by poor communication of results put them off re-attending screening, sometimes permanently:[10, 29, 88]

> *"She only said I had abnormal cells and that I could get cancer. She didn't explain anything and only caused me fear. I didn't go get checked for many years."* (Wittenberg et al., 2015, cervical, US)[88]

> *"Male doctors make mistakes. That's why I don't like to go to them (interpretation of mammography screening)! I like to see them talk to somebody else before they bring you back in there and scare you half to death. They talk over your head; they tell you things like ask your doctor. That's why I won't go back and get another one."* (Fowler 2006, breast, US)[10]

Recalls also created a lot of anxiety,[25, 36, 85] especially if participants had to wait for a follow-up appointment:[64, 85]

> *"You go through all [these] sleepless nights. Is it or isn't it, do I have this? I think about that because my mother had breast cancer and now my sister has been treated for breast cancer and she's younger than I am. So, the recalls are traumatic. I try to tell myself, "Look, you've been through this several times and it always has turned out okay, and after all, breast cancer is treatable, you know." I try not to worry about it, but I still do."* (Greco et al., 2010, breast, US)[36]

> *"They send me a letter on Friday, where it says that it was abnormal. And so I thought, OK, I am going to die. Whole weekend was so, because they said in the letter, go ahead and schedule a sonogram at your earliest convenience. They were closed; I had to wait until Monday."* (Engelman et al., 2012, breast, US)[85]

> [One] woman described her recall experience as follows: *"That is scary . . . I think that was the first time that they had to do more pictures—and then to be called back for an ultrasound. Then I had to wait for 3 or 4 days for the doctor . . . very scary."* (Greco et al., 2010, breast, US)[36]

> [The participant had] received, conducted, and returned a completed fecal occult blood test kit […]. The results came back positive. [She] was very upset, not only by the test results, but also by the way her physician reportedly handled the situation. […] He reportedly did not schedule another follow-up appointment for 2 months. [She] was very scared and upset. She did not want to wait 2 months for a follow-up appointment. (woman, Brouse et al., 2003, CRC, US)[64]

Thus, some people use screening to reduce cancer fear, but the screening experience itself may also be fear-provoking, and this fear may be enhanced by how the results are delivered. Although our literature search was limited to the context of cancer screening, similar fears have been shown to also exist in the context of help-seeking for possible cancer symptoms. For example, many people report being worried about what the doctor might find.[94, 95]

Trusting that someone is intervening on your behalf.

A third strategy to allay cancer fear is to trust in someone whom you believe will guard against cancer on your behalf. In some studies, people delegated this responsibility to their doctor;[7, 10, 15, 26, 38, 48] lacking a doctor's recommendation for screening was sometimes reassuringly interpreted that there was no need to worry: *"I assume that the doctor knows better what has to be done. If s/he does not suggest that I have a breast exam or mammography, there must be no need for it."*[15]

More commonly, however, participants reduced their cancer fear by trusting that God would intervene on their behalf. Religion was invoked in about a quarter of all included studies.[2, 5, 6, 10-12, 14, 17, 19, 21, 24, 31, 33, 35, 46, 58, 78, 86, 90, 96-99] We found references to divine intervention in studies from many countries and cultures, and regardless of the type of religion. Religion was invoked in one of two ways. A minority of participants used religion to help cope with their fears about cancer,[12, 14, 19, 96-98] or to enhance the power that they themselves[14, 31, 35] or their doctors[12, 19, 38] were believed to have over cancer through God, for example, by keeping faith, praying, and trusting that God works through doctors:

> The power of prayer also helps individuals cope with their cancer fears and ambivalence. One participant expressed: *"If it was in some way I was to get breast cancer … I'm not claiming it … If you're a strong individual and you believe in God, and you keep your faith, you can get through anything".* (Cohen, 2009, breast, US)[14]

> Religious faith attenuated the women's fear and reduced the threat of the disease: *[…] "A person is always afraid of getting sick but the treatment or getting better is from God."* (Bener et al., 2002, breast, United Arab Emirates)[12]

> [Some women] mentioned that their relationship with God would help them through, and some cited religious beliefs as a source for hope: *"I feel like with God's will, I will be all right. The spiritual part is important too. Don't claim the negativity."* (Wood and Della-Monica, 2011, breast, US)[35]

> [The women] believed God could act through screening: *"Doctors are messengers from God for treatment." "Act and I will act with you,' said God."* These beliefs may have a positive effect on their screening practice. (Bener et al., 2002, breast, United Arab Emirates)[12]

> Many described how God worked through doctors to achieve positive outcomes." (Greiner et al., 2005, CRC, US)[19]

Others, however, invoked religion not just as a coping mechanism, but also used it to abdicate their own control over the threat of cancer by leaving their fate up to God:[2, 5, 6, 10-12, 17, 21, 27, 33, 46, 58, 78, 86, 90, 99]

> While women discussed how their faith in God helped alleviate fears about future uncertainties, disease and death, by "leaving it in God's hands", some of the women abdicated their own agency in preventing breast cancer: *"So I don't worry about [breast cancer screening] because only God can decide when it's my time to go."* (Peek et al., 2008, breast, US)[46]
>
> *"I refuse to take either one of those tests (Pap). I won't go that way. No way. I leave everything up to God."* (Guilfoyle et al., 2007, cervical, US)[11]

Some people viewed cancer as part of God's plan or test, and therefore saw their own role as one of acceptance and patience:[10, 12, 46, 78, 86, 90, 97]

> Participants expressed a belief that fate and Allah's will determine their destiny in life. One woman reported *"we believe that fate and Allah's will control our future in regards to [breast cancer] and other events in life, there is no need for screening."* The women who shared this belief tended to see no benefit from [breast cancer screening] due to the fact that they believed in Allah's sovereign and ultimate will. Their beliefs lead them to state that the end result would be the same whether they had participated or not participated in [breast cancer screening]. As reported *"why to participate? If Allah's wills to get [breast cancer] mammography will not make a difference."* They indicated that they would accept that getting [breast cancer] was, somehow, part of Allah's plan and they believed that if this is Allah's will they could and perhaps should do nothing to stop it. As stated *"If Allah allows it I have to accept it, it is my fate."* (Kawar 2013, breast, US)[78]

Putting your trust in doctors or in a higher power seemed to allay cancer fear, but was also associated with lower motivation to attend screening in those who believed that events in life were predetermined by a higher power, a concept known as 'religious fatalism'.[100]

Ignoring the enemy.

A fourth strategy to deal with the threat of cancer was to ignore the existence of cancer altogether. Participants in more than half of included studies acknowledged their susceptibility to cancer, but preferred to ignore the threat of cancer because they could not deal with the fear associated with it.

A diagnosis was greatly feared by these participants, and statements such as "*ignorance is bliss"*[48, 90] and *"what you don't know you don't worry about"*[18, 42, 63, 101, 102] were used in more than a third of all included studies to explain why they did not participate in screening:[1, 3, 6, 10, 12, 13, 18, 19, 21, 24, 26, 34, 42, 48, 51, 63, 78, 82, 88, 90, 92, 101-115]

> *"I remember the first invitation, I threw it out....I just thought it was – I didn't want to know about it. I more or less got frightened and thought what I don't know won't bother me."* (woman, Woodrow et al., 2008, CRC, UK)[102]
>
> *"A lot of people are afraid to go to the doctor because of what they might find out, and then they won't go, 'cause they don't want to find out…"* (gender unknown, Jilcott Pitts et al., 2013, CRC, US)[115]
>
> The second most common reason for non-attendance was fear of being diagnosed with cancer […]: *"I am afraid of the screening results. If it is bad news and I would rather not know about it."* (female, Lee et al., 2014, breast, cervical, and stomach, Republic of Korea)[106]
>
> [One] women reported that fear of having cervical cancer prevented her from wanting a cervical screening test. If she knew that she had cancer, she would change as a person and shut down: *"To find out that I have some kind of illness... like to know you're sick you just want to block it out… If I*

knew I had an illness… I would probably just shut down from everything and totally change, and not for the better either." (O'Brien et al., 2009, cervical, Canada)[92]

Most people did not want to know about cancer, specifically, but some seemed to avoid bad news in general:[7, 11, 12, 18, 19, 31, 33, 38, 47, 48, 62, 78, 82, 104, 106, 108, 109]

> *"I'll call it the fear factor… do I really want to know that there's something wrong?"* (male, Ritvo et al., 2013, CRC, Canada)[18]
>
> *"Just finding out that something may be wrong is definitely the biggest fear of all."* (Kahn et al., 1999, cervical, US)[62]
>
> *"I feel afraid when I have no disease and have an examination, after that, they will tell me that I have a disease. It is better if they do not discover it."* (Bener et al., 2002, breast, United Arab Emirates)[12]
>
> Participants expressed *"fear of finding any minor breast irregularities, such as a lump or mass, even if it turned to be benign."* (Kawar, 2013, breast, US)[78]
>
> *"Fear of finding out something's wrong, fear of finding out they have an STD [sexually transmitted disease] or something's abnormal. Some people can't deal with stuff like that because they feel nasty. And they blame it on themselves."* (Kahn et al., 1999, cervical, US)[62]

Those who chose to deal with the threat of cancer by ignoring it seemed to prioritise feeling good or normal over the relative advantages of early detection:[11, 18, 19, 42, 50, 54, 58, 65, 78, 79, 81, 82, 101]

> Women frequently endorsed fear as an affective barrier to screening: *"Fear. Some people, they feel good and don't want to know if anything is wrong."* (Guilfoyle et al., 2007, cervical, US)[11]
>
> *"I have this terrible thing (idea) that … it'll kill you anyway….it'll get you in the end… so I'd rather not [get screened] now and live my life the way I do…"* (male, Ritvo et al., 2013, CRC, Canada)[18]
>
> *"If I am worrying whether or not I have [cancer], I'll stop living the life I have to live something else. That something [cancer], if it comes, it comes. Fortunately, I'm in good health."* (male, Molina-Barcelo et al., 2011, CRC, Spain)[50]

They avoided screening tests because they considered them as taking an unnecessary risk of finding cancer,[3, 12, 13, 21, 22, 48, 62, 65] almost as if the test could spontaneously make cancer appear: *"I have this fear that if I check for it and I find something then my life is gonna change. But […] if I don't check, I don't find anything and nothing changes."*[65] Quantitative studies have shown that the belief that going for a screening test would be "tempting fate" is a deterrent to cancer screening uptake, and that this belief is equally held across genders, but may be more prevalent among certain ethnic groups.[116, 117] The belief that screening forms an unnecessary risk of finding cancer may originate from wider fears about harms from medical intervention in general:[10, 42]

> [One participant] doubted whether screening would prevent serious consequences. A close friend had died of bowel cancer in spite of four operations. He knew it was not rational to assume that treatment was useless but the experience had affected his attitude to screening: *"[…] I know there are remarkable successes with the early treatment of bowel cancer but my friend did suffer tremendously through a series of operations that he had for his cancer. And I'm very proud of him, he was great, but as I said I think sometimes you tend to switch off and think, well, I'll have another pint and worry about it tomorrow."* (male, Chapple et al., 2008, CRC, UK)[42]

Finally, in about a sixth of included studies, participants ignored the threat of cancer because it competed with more pressing worries, such as other health problems,[26, 50, 65] illness of a close family

member,[54] or struggles to meet the many demands of everyday life:[1, 13, 15, 24, 32, 48, 50, 53, 54, 60, 63, 65, 88, 115, 118]

> *"I like, I've got enough to deal with my, with my MS [multiple sclerosis], like I don't want anything else to deal with. Let me do my own thing and just not have to worry about anything."* (Todd and Stuifbergen, 2011, breast, US)[65]
>
> *"I don't want to know if I have anything because my husband is already sick. I don't want to add to my family's worries."* (female, Bastani et al., 2001, CRC, US)[54]
>
> The daily responsibilities […] often took precedence in women's lives over attending to their own health and pursuing screening. […]: *"[…] you kind of like put yourself last. You know, you worry about your children. Then you worry about your husband. And then you worry about yourself. […]"* (female, Tkatch et al., 2014, cancer screening, US)[13]

Those from poorer or immigrant backgrounds, and those in developing countries indicated that worries about meeting the basic needs in life often took precedence over attendance at cancer screening, because they had "*no time for potential troubles*",[15] as also exemplified in the following quote: *"These countries are so poor that they don't worry about any of that [early detection of breast cancer]".*[24] These findings closely mirror findings from quantitative studies: for example, a large population-based survey in the UK reported that about a sixth (18%) of adults had other things to worry about which would serve as a barrier to seeking help for a possible cancer symptom.[119] This suggests that having competing worries can be a significant barrier to early detection of cancer, and may ultimately help explain ethnic and SES differences in cancer outcomes.

4. What if the enemy attacks?

All the studies in the review were conducted in healthy populations, yet many participants expressed fears about what it would mean to be diagnosed with cancer. Fears could be subdivided into fears about the emotional and physical consequences, and the social consequences of receiving a cancer diagnosis.

Emotional and physical implications of being a cancer patient.

In more than half of all included studies, participants talked about their fears of becoming a cancer patient because of the emotional turmoil this would cause, and the pain and suffering associated with the disease and its treatments.

Fears about the emotional implications of a cancer diagnosis were mentioned in a quarter of included studies and revolved around how to *"handle"* a cancer diagnosis, with participants expecting to feel devastated, anxious, sad and depressed.[2-4, 6-8, 10, 12, 13, 21, 24, 25, 33-35, 39, 41, 58, 78, 87, 88, 90, 92, 97, 98, 104, 120]

> Fear of possible CRC diagnosis as a result of the [screening] procedure was also a strong barrier to colonoscopy […]: *"I'm afraid to find out because I don't know how I'm going to deal with it."* (female, Hennelly et al., 2015, CRC, US)[41]
>
> *"[A diagnosis of breast cancer in my son's stepmother] brought up a lot of fear for me because I don't know if I could handle that."* (Purtzer and Overstreet, 2014, breast, US)[39]
>
> Anxiety, worry, and fear were also expressed when participants were asked what they thought might happen if they were diagnosed with breast cancer. […]:
> *"I think I would be devastated. […]"*
> *"I just think I would freak out […]"*
> *"I would be so afraid if I get it […]"*
> (Wood and Della-Monica, 2011, breast, US)[35]

> On being asked about how women […] would feel if [they] were diagnosed with breast cancer, almost unanimously they expressed feeling threatened […]:
> *Participant 1: "…I am terrified of that illness. Terrified! … Ehhh, and if I were diagnosed with cancer, I would feel very… just to be waiting for that pain. I would be worried."*
> *Participant 2: "Worried, yes. […] … worry, lots of worry."*
> […]
> Women were afraid that if they discovered that they have breast cancer, a series of negative events would unfold in their lives. For example, they anticipated psychological turmoil, such as feeling depressed, anxious, and worried from knowing that they had an illness that might ultimately be deadly […]: *"Depression. […] [One] gets depressed thinking that, well, believing that it is the end. […]"* (Borrayo, et al., 2005, breast, US)[21]

Participants worried that a diagnosis of cancer would make their world fall apart:[25, 58] *"You can go in there [i.e. screening] thinking nothing is wrong and come out with your whole life being changed."*[58]

Fears about the physical implications of being a cancer patient had to do with the disease itself, or with its treatments. Participants in several studies feared that by the time cancer was detected, it would already have spread to other parts of the body and that it would be *"too late"* to do anything about it:[5, 10, 19, 34, 39, 90, 115]

> [Most of the women] mentioned [cancer's] spread to the body if its diagnosis is delayed […] as a source of fear. […] One woman said: *"If treatment fails, cancer invades the whole body. Suffering due to this invasion is dreadful. […]"* Another woman said, *"I'm only worried that it can spread to the whole body, which is dangerous. No way."* (Kissal and Beşer, 2011, breast, Turkey)[5]

> *"[My son's stepmother's diagnosis of breast cancer] brought up a lot of fear for myself because the first thing I thought is, "My God, it spread to the lymph nodes and it's pretty serious."* (Purtzer and Overstreet, 2014, breast, US)[39]

> *"Cancer is a scary thing, period. By the time it's detected it spreads all through your body. It's too late."* (Fowler, 2006, breast, US)[10]

More often, however, fears had to do with the treatments for cancer. Fears about cancer treatment were mentioned in more than 40 studies.[4-6, 8, 10, 11, 14-16, 19, 21, 22, 24, 27, 31, 32, 34, 35, 38-42, 45, 46, 50, 78, 79, 87, 90, 92, 93, 97-99, 101, 104, 115, 121-123] Participants feared pain, suffering, and bodily changes, such as hair loss, emaciation, and resection scars, associated with cancer treatments:[3, 5, 6, 8, 11, 14, 21, 24, 27, 37, 41, 46, 50, 78, 79, 90, 124]

> *"I have no fear of death, I am afraid of pain, suffering, and causing suffering to those around me."* (female, Molina-Barcelo et al., 2011, CRC, Spain)[50]

> *"The fear of just finding cancer. I think that scares a lot of people. Fear about pain and suffering."* (gender unknown, Greisinger et al., 2006, CRC, US)[37]

> Several White male focus group participants were concerned that finding cancer meant undergoing painful treatments and extending a life of pain. (Holmes-Rovner et al., 2002, CRC, US)[79]

> Women feared the consequences of having [breast cancer] and its subsequent treatments […]: *"Fear of finding the disease. Fear of […] the body changes that take place after [chemotherapy]."* (Kawar, 2013, breast, US)[78]

> […] all of the Latinas in this study also expected that if they were diagnosed with breast cancer, they would risk experiencing various physical consequences, such as being sick, bleeding, feeling pain, discomfort, and disfigurement. […]: *"I would feel very bad because of the pain [that I would] have, the discomfort, the burning . . ."* (Borrayo et al., 2005, breast, US)[21]

Chemotherapy[14, 41, 78, 121] and surgery[4-6, 8, 14, 16, 19, 21, 22, 24, 27, 31, 32, 34, 35, 38, 39, 42, 45, 46, 83, 90, 92, 93, 98, 99, 101, 104] were often mentioned as particular fears, and partly stemmed from encounters with cancer in others (see also theme 2: 'Cancer encounters'):

> *"I'm afraid that they'll find something. My stupid thinking is, "Well, if I have it, what are they going to do? I don't want chemotherapy."* (Friedman et al., 2012, breast and cervical, US)[121]
>
> The fear that diagnosis would require surgery was one reason women reported avoiding screening. (Cohen, 2009, breast, US)[14]
>
> Women feared the consequences of having [breast cancer] and its subsequent treatment: […] *"It is hard to watch someone who had [breast cancer] go through treatment, and watch the consequences of treatment."* (Kawar, 2013, breast, US)[78]
>
> *"I saw a woman who had a large scar on her left chest in a public sauna in Korea. It was so bizarre to me. Her scar was on her left chest from shoulder to middle of the chest. […] I felt so bad for that woman. Cancer is so fearful for me since I saw her scar."* (Suh, 2006, breast, US)[8]

Fear of cancer surgery was also expressed as a fear of having body parts cut off and deformed, particularly if this would be visible to others:[4, 6, 14, 21, 24, 27, 31, 39, 101]

> [Participants] expressed fear of actually being diagnosed with colon cancer and that they would prefer not to know. […]: *"[…] If I ever had it, I would never let them cut me or anything like that… nothing."* (female, Green et al., 2008, CRC, US)[101]
>
> *"[…] I would prefer to die and be buried in one piece than being cut and sold by kilo."* (Taha et al., 2012, breast, Jordan)[6]
>
> *"But for sure the first thing that one is going to think [on diagnosis] is to have it [the breast] cut...how am I going to look and what am I going to do ? I think that is something that makes you fearful."* (Borrayo et al., 2005, breast, US)[21]
>
> Some women fear that they will be disfigured if they get breast cancer: *""I am afraid. I don't want to have an operation. I wouldn't want to have one breast big and one breast small."* (Salazar, 1996, breast, US)[27]

Related to the social implications of being a cancer patient discussed below, some women expressed fears of losing intimate body parts with symbolic value. In some religions, the cervix is the place where a woman's soul resides,[92] while the breasts (and to a lesser extent the cervix) were widely considered to be symbols of femininity and motherhood.[4, 6, 21, 22, 24, 32, 83, 86, 92, 98, 99] Losing these body parts was therefore likened to losing your gender identity:

> For many of our informants, the breast was not simply an organ, but a symbol of femininity. Therefore they saw breast cancer as not only threatening their lives, but also their womanhood, since most believed that breast cancer would always lead to the removal of the breasts. […] Because breast cancer was seen as a ''lady-killer'', informants were doubly fearful of the disease. Typical comments were: *"If I lose my breasts, I will no longer feel I'm a woman"* and *"I think breast cancer is the most scary disease for a woman because it takes away the breasts."* (Kwok et al., 2006, breast, Australia)[83]
>
> In all the [focus groups], women associated breast cancer with fear of a distorted body image and loss of femininity because it inflicts a body organ that symbolises femininity and motherhood: *"A women who gets breast cancer will be devastated; since losing her breasts means that she is finished as a woman and as a mother."* (Taha et al., 2012, breast, Jordan)[6]

> [One] discussion topic […] was fear of mastectomy. One participant described the idea of having [a] mastectomy as *"you are less of a woman".* (Fernandez et al., 2005, breast, US)[32]

> [One] fear [women had] was losing their breast because they felt that they would also lose a symbol of their own femininity and pride. (Buki et al., 2004, breast, US)[24]

Some people's fear of surgery was caused by the belief that cutting cancer causes it to spread, and so the need for cancer surgery would present an impossible decision: [19, 34, 46, 104]

> Virtually all focus group participants had heard people say that cancer spreads when a patient is opened up and "air hits the cancer". Although the majority of our participants did not endorse this belief, they described it as a very common belief in their community:
> *"Well, you know some older people that think that once you find cancer and start cutting on cancer, cancer will spread. At the first sign of cutting on it, the cancer will spread through your body."*
> *"But you also hear the rumours where people have been opened up and it seemed like when they were opened up, the cancer spread. And I think that is my biggest fear."*
> (genders unknown, Greiner et al., 2005, CRC, US)[19]

> Some women feared having unnecessary surgery or mastectomy. Some of this fear of "wrongful surgery" was due to […] the well-documented myth that surgery causes cancer to spread (and would therefore be unnecessary or harmful) was prevalent among this group of African-American women, despite health education efforts over the prior decades to eradicate such beliefs. (Peek et al., breast, US)[46]

The belief that cancer spreads when operated on, or when "air hits the cancer", may be more prevalent among certain population subgroups, and was associated with late stage presentation of breast cancer in a study from the 1990s.[125] Future studies should address whether this belief is still prevalent in the population, and to what extent these worries forms a barrier to uptake of cancer screening.

Fears about cancer treatments may be exacerbated by the perception that they cannot provide a long-term cure,[6, 8, 10, 11, 13-16, 19, 40, 42, 87, 90, 97] or that they equate to torture and that it would be better to die than undergo cancer treatment:[15, 50]

> *"My aunt had breast cancer […]; she had access to all the best cancer therapy facilities of the city, and for two years she received two surgeries, chemotherapy, and irradiation non-stop. She got all exhausted and emaciated, lost all her hair, couldn't eat… and what in the end? She died after these two and a half years of torture. I think that if she hadn't have this tumor discovered early, she could have spent these two years much more happily and have died quickly when cancer finally got a total grasp of her body. I am not convinced that early diagnosis can really save your life."* (Remennick, 2003, breast, Israel)[15]

> We identified other reasons for not participating [in cancer screening] in the individual discourses […]. [One] man said he preferred a quick death without having to deal with hospitals and physicians: *"I prefer a quick death than passing through 40 000 hands. I'm sure."* (male, Molina-Barcelo et al., 2011, CRC, Spain)[50]

> *"She made this big announcement about how: ladies, we have to screen ourselves, how she screened herself, and thank God they caught it, and she's here to tell the story that thank God she's alive because of screening. And she died like two years later of breast cancer."* (female, Tkatch et al., 2014, cancer screening, US)[13]

The studies that made a link with screening uptake mostly reported that fears of treatment would lead to screening avoidance,[4, 5, 14, 15, 19, 21, 24, 38, 41, 42, 45, 46, 50, 78, 79, 87, 93, 101, 104, 115, 121, 123, 126] although there were some exceptions:[4, 5, 39, 41, 50, 86]

> Fear of cancer disease-related suffering was also found to be a strong facilitator to colonoscopy adherence […]. One adherent male explained: *"It's better to know than to not know because if you wait too late, then you're going to have to go through expensive chemo. I've seen what my brother goes through [with his cancer treatment], and I'm not going to go through that."* (male, Hennelly et al., 2015, CRC, US)[41]

> [W]omen admitted that their participation [in colorectal cancer screening] was out of fear of developing the disease because they wanted to avoid personal and family suffering. (Molina-Barcelo et al., 2011, CRC, Spain)[50]

Social implications of being a cancer patient.

People in about one third of included studies talked about their fears of what it means to be a cancer patient in a social sense, such as other people's reactions to a cancer diagnosis, and the effect it would have on their families and on their own social standing.

Fears about others' reactions were mentioned in about half of these studies, and included a fear of being stigmatised or blamed for having caused their own cancer,[2, 3, 6, 16, 19, 22, 27, 28, 30, 62, 66, 78, 83, 86, 98, 123] because some believed cancer to be a punishment for sins, or bad karma:[2, 3, 16, 83, 86]

> A possible subsequent diagnosis of breast cancer was greatly feared. The women indicated that cancer was one of a few health issues which were still considered taboo in Barbados […] and that this fear of a negative reaction from others was a barrier to screening:
> *"People are still ashamed, they aren't coming in early, and they are scared about the reaction that they will get from other people."*
> *"Fear of being stigmatised with cancer, because others found out. Barbados is small and we all know each other in some way. Because some people are ignorant and will shy away from a person with cancer."*
> *"It's like a stigma, people say 'oh do not talk to her', it's like the stigma with AIDS, if you have AIDS you won't say anything."*
> (Granado et al., 2014, breast, Barbados)[98]

> A few of the ethnic minority women talked about potential shame if diagnosed with cervical cancer as a barrier to attending screening among others, particularly the older generation: *"they are worried about what will people say, why did I get it, how did I get it, questions will be raised and I will have to feel ashamed."* (Marlow et al., 2015, cervical, UK)[30]

> [Women] tended to view cancer as shameful and generally referred to cancer as a *"sign of shame"*. A participant reported *"cancer is a sign of shame"*. […] There was shame, as well as fear, that if a woman was found to have [breast cancer]*"it would reflect badly on her" and "reflect badly on her family."* (Kawar, 2013, breast, US)[78]

> Religious beliefs also seem to create a sense of shame about breast cancer that results in a fear of social stigmatisation. […]
> *"If you get breast cancer others will say that she has done a lot of sins, that's why .... they say we have done a lot of sins and that's why she has breast cancer."*
> *"Some people are over religious. They say it is their Karma."* […]
> Purportedly because of this religious belief, social stigmatisation is feared and women report fear that if it becomes known that they are going for a mammogram people will think they already have breast cancer. (Meana et al., 2001, breast and cervical, Canada)[2]

Cervical and breast cancer were sometimes associated with promiscuous behaviour and the thought of having to reveal this type of diagnosis provoked shame and fears of social rejection and gossip.[16, 28, 83, 86] For this reason, some preferred to keep a diagnosis of cancer secret:

> Given the close link they saw between women's sexuality and their breasts, some [women] believed that breast cancer is a particularly shameful disease when compared to other types of cancer because it can imply that the victim has been guilty of immoral behaviour. […]:
> *"I find it [breast cancer] shameful. People may think that you are immoral."*
> After further discussion, we discovered that the word ''immoral'' in this context was used as a synonym for promiscuity." (Kwok et al., 2006, breast, Australia)[83]

> Partly because of the association with "promiscuity", stigma was clearly associated with a diagnosis of [cervical] cancer in the Coloured and Zulu datasets. The perceived sexual aetiology of the illness meant that it was a "disgrace" […]: *"it is a disgrace because people have a tendency of sleeping with anyone, and they are afraid that it might have occurred because of that, so they can't tell other people."* (Wood et al., 1997, cervical, South Africa)[16]

> In addition the women discussed the fear of finding out you might have cancer, a stigma that creates an inability to talk about cervical cancer within the Hmong community. Stigma was also [associated] with having cervical cancer due to the community's perception that the women has engaged in inappropriate behaviour such as "sleeping around." (Fang and Baker, 2013, cervical, US)[28]

Fears about stigmatisation, social rejection and gossip seemed to lead to avoidance of screening,[3, 22, 28, 30, 62, 66, 78, 86, 98, 123] although one paper mentioned that the expectation of social rejection could motivate screening attendance *"to prevent this sooner and not later"*:[4]

> [One benefit of screening was to] avoid possible social rejection associated with diseases. […]: *"So it's better to prevent this sooner and not later, because when you're healthy, people great you or say, look how pretty she is, right? When you're sick, people stay away from you, out of disgust, they discriminate against you. So that's why you should take care of your own body, right?"* (Agurto et al., 2004, cervical, Venezuela, Ecuador, Mexico, El Salvador, Peru)[4]

About a sixth of included studies mentioned fears about the consequences of getting a cancer diagnosis on the immediate family, in terms of being a financial, physical, or psychological burden.[3-6, 14, 21, 24-26, 43, 50, 54, 66, 78, 90, 106, 115, 123] In a few studies, fear of burdening the family would prevent participants from sharing a cancer diagnosis with family members.[14, 24, 123]

> *"[A diagnosis of cancer] can be a burden on you… mentally, physically, financially… and a burden on your family when you're so sick."* […]
> "*It's […] very scary to not know if you can afford to take care of yourself, or if you're going to be a burden and drag down your whole family."* (females, Jones et al., 2010, CRC, US)[90]

> [Of great concern to participants] was the fear that their partners, children, siblings, and other extended relatives would go through psychological turmoil and would be worried, distressed, and anxious. […] *"I think that they [the family] become more worried than even the patient herself."* (Borrayo et al., 2005, breast, US)[21]

> Women […] feared that a diagnosis would have dire emotional and financial consequences for family members, therefore they would not tell their families if diagnosed. (Buki et al., 2004, breast, US)[24]

There were further fears that family members would also be affected by social rejection or stigmatisation,[6, 66, 78] which sometimes stemmed from the perceived heredity of cancer. Fears about family ramifications were linked to preferences for screening avoidance,[3, 4, 24, 54, 66, 78, 90, 106, 115, 123] although there were again some exceptions.[5, 50]

A recent study showed that out-of-pocket expenses incurred by patients who had free access to breast cancer care, for things such as food, lodging, transportation, childcare, and lost wages, in one lower-and-middle-income country forced many into debt, or to sell possessions.[127] Similarly, a study from a high-income country showed that in the US, cancer patients were at greater

risk of bankruptcy than people without a cancer diagnosis.[128] Thus, apart from the emotional aspects, the financial burden of a cancer diagnosis can indeed be considerable, and this prospect may negatively impact willingness to screen.

For women, fears about partner rejection and abandonment if diagnosed with breast or cervical cancer were a major concern. Women were not only worried that they would lose their femininity if they needed to undergo cancer treatment (see 'emotional and physical implications of a cancer diagnosis'), but also feared that a cancer diagnosis would jeopardise their social role and relationship with their partner. Cancer treatment (particularly for breast cancer) would leave them feeling incomplete, de-feminised, de-valued, unattractive, and sexually compromised,[6, 7, 22, 24, 32, 46, 78, 86, 99, 123] which they felt would render them worthless as a wife or mother, and they were afraid that their husbands might abandon them in favour of a woman without cancer:[6, 24, 32, 46, 78, 98]

> Some women reported having fears of downstream psychosocial sequelae that might arise from a diagnosis of breast cancer. The potential impact of a mastectomy on body image and interpersonal relationships was a commonly reported concern. For example, there was concern that having a mastectomy would precipitate abandonment by their partners […]:
> *"If I had to have [a breast] removed and my husband was still living with me… it would cause me to have concern. The first thing to come to mind is "Oh, he is going to get another woman now… I'm no longer attractive."* (Peek et al., 2008, breast, US)[46]
>
> [The] fear of a breast cancer diagnosis included possible emotional and social consequences of altered female bodies, for example, losing a romantic relationship as a result of surgery: *"For some women, the thought of losing a breast means that your boyfriend is not going to love you anymore, your husband is not going to want you. […] Our breasts define our sexuality, our femininity."* (Granado et al., 2014, breast, Barbados)[98]
>
> [Women] tended to mention effects [of cervical cancer] on family and sexual functions:
> *"It's said to affect your sexuality, yes. […]"*
> *"It'll certainly change between me and my husband. […]"*
> *"There'll be nothing left between me and my husband...it can be like that."*
> (Duran, 2011, cervical, Turkey)[7]
>
> [Women] were of the opinion that there are few men who would stand by their wife if she had breast cancer. In all [focus group discussions], women had observed that men whose wives had been stricken by breast cancer had started looking for other women […]: *"I know a woman who had breast cancer. Her husband rejected her and married another woman because she lost her femininity."* (Taha et al., 2012, breast, Jordan)[6]

Fears about the effect of a cancer diagnosis on intimate relationships were only expressed by women and in the context of breast and cervical cancer screening, but this may be due to the selection of screening programmes included in this review; it is possible that men have similar fears regarding cancer treatments that were not included in this review but which could be perceived to affect masculinity, e.g. for testicular or prostate cancer. Future studies should address the validity of the taxonomy of cancer fears presented here in men.

In addition, some fears may be more prevalent for certain types of cancer. For example, womanhood issues may be more prevalent for breast cancer than for colorectal cancer, and fears of being blamed or stigmatised may be more prevalent for lung cancer than for some other types of cancer. The aim of this review, however, was to explore and categorise the fears that cancer (in general) evokes in the general population, and not to explore differences in cancer fears between cancer types. Future studies should explore the prevalence of the various fears for different types of cancer.

5. And finally, what if the enemy wins?

The last theme consists of fears about mortality. 'Death' was an inherent meaning of cancer in more than a third of included studies, and this was a source of intense fear:[1, 2, 4, 6, 8, 14-16, 18, 19, 21, 24, 26, 27, 32, 34, 36, 37, 40, 41, 46, 49, 52, 63, 78, 83, 85-87, 90, 92, 97, 99, 106, 113, 115, 123, 124, 129]

> Many of the women were fearful of cancer because they felt that it was a "death sentence" and believed that once a person was diagnosed he or she would die: *"[…] it was a big fear for me because as soon as you hear cancer you think of the ultimate. There's nothing more then, but for this person to die."* (O'Brien et al., 2009, cervical, Canada)[92]
>
> Fear of cancer –not only colorectal cancer but cancer in general- was a major theme in all focus groups. The view that colorectal cancer was a fatal disease was repeatedly brought up as an issue: *"Just the word cancer frightens people. We just all assume you die of it."* (male, Tessaro et al., 2006, CRC, US)[49]
>
> Fear was a major barrier to CRC screening. […]: *" . . .When you heard the word cancer, you don't think of cancer, you think of death."* (gender unknown, Jilcott Pitts et al., 2013, CRC, US)[115]
>
> *"I think it scares a lot of people, the word 'cancer', because they think cancer is terminal."* (Kelly et al., 2015, cervical, US)[97]

Some participants had difficulty reconciling messages from doctors and the media about improved cancer survival with what they had experienced, or what they merely feared to be the case. This sometimes led to confusion:[11, 13-15, 29, 106]

> *"They say that you don't die from cancer and everyone does."* (Guilfoyle et al., 2007, cervical, US)[11]
>
> A cancer diagnosis was perceived by many informants as equal to death sentence, and their instinctive line of behaviour would be to distance themselves as far as possible from any cancer-related medical procedures. Although all of them have heard about the role of early detection for higher survival, this knowledge failed to transform their beliefs about this disease. […]: *"Most people I know who got cancer eventually died from it, and it was sooner rather than later. May be all of them got their diagnosis late, I don't know. But I've never heard of anyone with cancer who got cured"* (Remennick, 2003, breast, Israel)[15]
>
> *"I think cancer is such a scary word, because we don't see … I haven't seen a lot of people get past it. Those survivors, like you see them all running downtown is beautiful, wonderful stories, but close up, at least people … I speak for myself, that I know … don't survive it."* (Cohen, 2009, breast, US)[14]
>
> The second most common reason for non-attendance [at screening] was fear of being diagnosed with cancer, [which was] usually based on fatalistic views about cancer. Some participants believed that a cancer diagnosis inevitably leads to death, and they were therefore unsure as to why physicians would promote a screening test to detect an incurable disease. (Lee et al., 2014, breast, cervical, and stomach, Republic of Korea)[106]

Some said that it was better to die without knowing you had cancer,[3, 6, 18, 21, 24, 28, 32, 78, 87, 89, 97] suggesting that fear of dying is also fear of knowing you were going to die:

> Many [women] did not want to go to the doctor for fear that they will be told they will die. Because informants believed that breast cancer is fatal, they anticipated that knowledge of a breast cancer diagnosis would render them terribly hopeless:
> Participant: *"I'd rather not go, that way the doctor can't tell me that I have cancer, because just by knowing, that I will, that I will die, just from knowing . . . if I go to the doctor tomorrow and he tells me "You have breast cancer," I would die."*

> Interviewer: *"You would die from knowing or . . ."*
> Participant: *"From knowing. From the fear"*
> (Buki et al., 2004, breast, US)[24]
>
> *"Also, [a barrier to screening] is fear. You don't want to go find out that you're only going to live for how many months or so."* (Fang and Baker, 2013, cervical, US)[28]
>
> *"I have this terrible thing (idea) that… it'll kill you anyway… it'll get you in the end… so I'd rather not [get screened] now and live my life the way I do…"* (male, Ritvo et al., 2013, CRC, Canada)[18]
>
> [Some participants had a] perception that early detection would not be useful, and that the emotional turmoil of knowing one having a terminal disease would only bring about more stress and worry. (Wong et al., 2008, cervical, Malaysia)[3]

This fear of knowing you are going to die from cancer may be related to the *'ignorance is bliss'*-type of dealing with the cancer threat mentioned earlier (see theme 3 'Ignoring the enemy').

Others feared that knowing could make you die sooner or would cause the cancer to progress, as if the inevitability would somehow impel you towards death:[21, 24, 87, 89]

> Some women […] said that if they ever developed breast cancer, they would rather not know that they have it. For instance, [one woman] recalled telling her doctor, *"No, no, no, I don't want to know [if I have breast cancer]…. "ay," he told me" [participant's name], but what a coward you are." I told him, "but doctor I can't know this, that I am in a critical condition, because I will die sooner."* (Borrayo et al., 2005, breast, US)[21]
>
> Many [women] cited fear of […] positive test results [of cervical cancer screening], and thought that knowing they had cervical cancer would result in an early death. (Busingye et al., 2012, cervical, Uganda)[89]
>
> Women acknowledged a sense of fatalism regarding cancer and felt that 'knowing their status' would be a situation too difficult to cope with. In addition, they did not want to worry about the outcome. They felt that worrying would make the disease worse. (Teng et al., 2014, cervical, Uganda)[87]

The high prevalence of specific fears about mortality in studies about screening suggests that the association of cancer with death is near-universal, cross-cultural and, together with fears about the implications of being a cancer patient, perhaps the most important component of cancer fear.

Abbreviations

CRC	Colorectal cancer
HINTS	Health Information National Trends Survey
UK	United Kingdom
US	United States

References§

§Reference numbers in this supplement do not match those of the manuscript.

* 1. Ishida DN, Toomata-Mayer TF, Braginsky NS. Beliefs and attitudes of Samoan women toward early detection of breast cancer and mammography utilization. *Cancer*. 2001;**91**:262-266.

* 2. Meana M, Bunston T, George U, Wells L, Rosser W. Older immigrant Tamil women and their doctors: attitudes toward breast cancer screening. *J Immigr Health*. 2001;**3**:5-13.

* 3. Wong LP, Wong YL, Low WY, Khoo EM, Shuib R. Cervical cancer screening attitudes and beliefs of Malaysian women who have never had a pap smear: A qualitative study. *Int J Behav Med*. 2008;**15**:289-292.

* 4. Agurto I, Bishop A, Sánchez G, Betancourt Z, Robles S. Perceived barriers and benefits to cervical cancer screening in Latin America. *Prev Med*. 2004;**39**:91-98.

* 5. Kissal A, Beşer A. Knowledge, facilitators and perceived barriers for early detection of breast cancer among elderly Turkish women. *Asian Pac J Cancer Prevent*. 2011;**12**:975-984.

* 6. Taha H, Al-Qutob R, Nyström L, Wahlström R, Berggren V. "Voices of Fear and Safety" Women's ambivalence towards breast cancer and breast health: A qualitative study from Jordan. *BMC Womens Health*. 2012;**12**:21.

* 7. Duran ET. Examination with the health belief model of women's attitudes to cervical cancer and early diagnosis in Turkey: A qualitative study. *Asian Pac J Cancer Prev*. 2011;**12**:1179-1184.

* 8. Suh EE. Korean immigrant women's meanings of breast, breast cancer, and breast cancer screenings. *Taehan Kanho Hakhoe Chi*. 2006;**36**:604-611.

* 9. Willis K, Baxter J. Trusting technology: women aged 40-49 years participating in screening for breast cancer--an exploratory study. *Aust N Z J Public Health*. 2003;**27**:282-286.

* 10. Fowler BA. Social processes used by African American women in making decisions about mammography screening. *J Nurs Scholarsh*. 2006;**38**:247-254.

* 11. Guilfoyle S, Franco R, Gorin SS. Exploring older women's approaches to cervical cancer screening. *Health Care Women Int*. 2007;**28**:930-950.

* 12. Bener A, Honein G, Carter AO, Da'ar Z, Miller C, Dunn EV. The determinants of breast cancer screening behavior: a focus group study of women in the United Arab Emirates. *Oncol Nurs Forum*. 2002;**29**:E91-98.

* 13. Tkatch R, Hudson J, Katz A, et al. Barriers to cancer screening among Orthodox Jewish women. *J Community Health*. 2014;**39**:1200-8.

* 14. Cohen EL. Naming and claiming cancer among African American women: an application of Problematic Integration Theory. *J Appl Commun Res*. 2009;**37**:397-417.

* 15. Remennick L. "I have no time for potential troubles": Russian immigrant women and breast cancer screening in Israel. *J Immigr Health*. 2003;**5**:153-163.

* 16. Wood K, Jewkes R, Abrahams N. Cleaning the womb: constructions of cervical screening and womb cancer among rural black women in South Africa. *Soc Sci Med*. 1997;**45**:283-294.

* 17. Scarinci IC, Litton AG, Garcés-Palacio IC, Partridge EE, Castle PE. Acceptability and usability of self-collected sampling for HPV testing among African-American women living in the Mississippi Delta. *Womens Health Issues*. 2013;**23**:e123-e130.

* 18. Ritvo P, Myers RE, Paszat L, Serenity M, Perez DF, Rabeneck L. Gender differences in attitudes impeding colorectal cancer screening. *BMC Public Health*. 2013;**13**:500.

* 19. Greiner KA, Born W, Nollen N, Ahluwalia JS. Knowledge and perceptions of colorectal cancer screening among urban African Americans. *J Gen Intern Med*. 2005;**20**:977-983.

20. Clarke JN, Everest MM. Cancer in the mass print media: fear, uncertainty and the medical model. *Soc Sci Med*. 2006;**62**:2591-2600.

* 21. Borrayo EA, Buki LP, Feigal BM. Breast cancer detection among older Latinas: is it worth the risk? *Qual Health Res*. 2005;**15**:1244-1263.

* 22. Khazaee-Pool M, Majlessi F, Foroushani AR, et al. Perception of breast cancer screening among Iranian women without experience of mammography: a qualitative study. *Asian Pac J Cancer Prev*. 2014;**15**:3965-3971.

* 23. Naish J, Brown J, Denton B. Intercultural consultations: investigation of factors that deter non-English speaking women from attending their general practitioners for cervical screening. *BMJ*. 1994;**309**:1126-1128.

* 24. Buki LP, Borrayo EA, Feigal BM, Carrillo IY. Are all Latinas the same? Perceived breast cancer screening barriers and facilitative conditions. *Psychol Women Q*. 2004;**28**:400-411.

* 25. Nekhlyudov L, Ross-Degnan D, Fletcher SW. Beliefs and expectations of women under 50 years old regarding screening mammography: a qualitative study. *J Gen Intern Med*. 2003;**18:**182-189.

* 26. Beeker C, Kraft JM, Southwell BG, Jorgensen CM. Colorectal cancer screening in older men and women: qualitative research findings and implications for intervention. *J Community Health*. 2000;**25**:263-278.

* 27. Salazar MK. Hispanic women's beliefs about breast cancer and mammography. *Cancer Nurs*. 1996;**19**:437-446.

* 28. Fang DM, Baker DL. Barriers and facilitators of cervical cancer screening among women of Hmong origin. *J Health Care Poor Underserved*. 2013;**24**:540-555.

* 29. Kaltsa A, Holloway A, Cox K. Factors that influence mammography screening behaviour: a qualitative study of Greek women's experiences. *Eur J Oncol Nurs*. 2013;**17**:292-301.

* 30. Marlow LA, Waller J, Wardle J. Barriers to cervical cancer screening among ethnic minority women: a qualitative study. *J Fam Plann Reprod Health Care*. 2015;**41**:248-254.

* 31. Tejeda S, Thompson B, Coronado GD, Martin DP. Barriers and facilitators related to mammography use among lower educated Mexican women in the USA. *Soc Sci Med*. 2009;**68**:832-839.

* 32. Fernandez ME, Palmer RC, Leong-Wu CA. Repeat mammography screening among low-income and minority women: a qualitative study. *Cancer control* . 2005;**12(Suppl 2)**:77-83.

* 33. Lee MC. Knowledge, barriers, and motivators related to cervical cancer screening among Korean-American women: a focus group approach. *Cancer Nurs*. 2000;**23**:168-175.

* 34. Wood RY, Della-Monica NR. Promoting breast health: older women's perceptions of an innovative intervention to enhance screening. *Int J Older People Nurs*. 2006;**1**:75-84.

* 35. Wood RY, Della-Monica NR. Psychosocial factors influencing breast cancer risk appraisal among older women. *Qual Health Res*. 2011;**21**:783-795.

* 36. Greco KE, Nail LM, Kendall J, Cartwright J, Messecar DC. Mammography decision making in older women with a breast cancer family history. *J Nurs Scholarsh*. 2010;**42**:348-356.

* 37. Greisinger A, Hawley ST, Bettencourt JL, Perz CA, Vernon SW. Primary care patients' understanding of colorectal cancer screening. *Cancer Detect Prev*. 2006;**30**:67-74.

* 38. Trigoni M, Griffiths F, Tsiftsis D, Koumantakis E, Green E, Lionis C. Mammography screening: views from women and primary care physicians in Crete. *BMC Womens Health*. 2008;**8**:20.

* 39. Purtzer MA, Overstreet L. Transformative learning theory: facilitating mammography screening in rural women. *Oncol Nurs Forum*. 2014:**41**;176-184.

* 40. Sly JR, Edwards T, Shelton RC, Jandorf L. Identifying barriers to colonoscopy screening for nonadherent African American participants in a patient navigation intervention. *Health Educ Behav*. 2013:**40**;449-457.

* 41. Hennelly MO, Sly JR, Villagra C, Jandorf L. Narrative message targets within the decision-making process to undergo screening colonoscopy among Latinos: a qualitative study. *J Cancer Educ*. 2015;**30**:268-276.

* 42. Chapple A, Ziebland S, Hewitson P, McPherson A. What affects the uptake of screening for bowel cancer using a faecal occult blood test (FOBt): a qualitative study. *Soc Sci Med*. 2008;**66**:2425-2435.

* 43. Ogedegbe G, Cassells AN, Robinson CM, et al. Perceptions of barriers and facilitators of cancer early detection among low-income minority women in community health centers. *J Natl Med Assoc*. 2005;**97**:162-170.

* 44. Ealey J, Meade CD, Gwede CK, et al. Patients' perspectives on immunochemical fecal occult blood test (I-FOBT or FIT): Not your father's FOBT. *Cancer Epidemiol Biomarkers Prev*. 2011;**20(10 Supplement)**:A9.

* 45. Jackson JC, Taylor VM, Chitnarong K, et al. Development of a cervical cancer control intervention program for Cambodian American women. *J Community Health*. 2000;**25**:359-375.

* 46. Peek ME, Sayad JV, Markwardt R. Fear, fatalism and breast cancer screening in low-income African-American women: the role of clinicians and the health care system. *J Gen Intern Med*. 2008;**23**:1847-1853.

* 47. Varela A, Jandorf L, Duhamel K. Understanding factors related to Colorectal Cancer (CRC) screening among urban Hispanics: use of focus group methodology. *J Cancer Educ*. 2010;**25**:70-75.

* 48. Wackerbarth SB, Peters JC, Haist SA. "Do we really need all that equipment?": factors influencing colorectal cancer screening decisions. *Qual Health Res*. 2005;**15**:539-554.

* 49. Tessaro I, Mangone C, Parkar I, Pawar V. Knowledge, barriers, and predictors of colorectal cancer screening in an Appalachian church population. *Prev Chronic Dis*. 2006;**3**:A123.

* 50. Molina-Barceló A, Salas Trejo D, Peiró-Pérez R, Málaga López A. To participate or not? Giving voice to gender and socio-economic differences in colorectal cancer screening programmes. *Eur J Cancer Care*. 2011;**20**:669-678.

* 51. Ekberg M, Callender M, Hamer H, Rogers S. Exploring the decision to participate in the National Health Service Bowel Cancer Screening Programme. *Eur J Cancer Prev*. 2014;**23**:391-397.

* 52. Ersin F, Bahar Z. Barriers and facilitating factors perceived in Turkish women's behaviors towards early cervical cancer detection: a qualitative approach. *Asian Pac J Cancer Prev*. 2013;**14**:4977-4982.

* 53. Percac-Lima S, Bond B, Saadi A. Bosnian, Iraqi, and Somali refugee women speak: a comparative study of refugee health beliefs on preventive health and breast cancer screening. *J Gen Intern Med*. 2013:S35-S35.

* 54. Bastani R, Gallardo NV, Maxwell AE. Barriers to colorectal cancer screening among ethnically diverse high- and average-risk individuals. *J Psychosoc Oncol*. 2001;**19**:65-84.

55. Wardle J, Sutton S, Williamson S, et al. Psychosocial influences on older adults' interest in participating in bowel cancer screening. *Prev Med*. 2000;**31**:323-334.

56. de Wijkerslooth TR, de Haan MC, Stoop EM, et al. Reasons for participation and nonparticipation in colorectal cancer screening: a randomized trial of colonoscopy and CT colonography. *Am J Gastroenterol*. 2012;**107**:1777-1783.

* 57. Royak-Schaler R, Blocker DE, Yali AM, Bynoe M, Briant KJ, Smith S. Breast and colorectal cancer risk communication approaches with low-income African-American and Hispanic women: implications for healthcare providers. *J Natl Med Assoc*. 2004;**96**:598-608.

* 58. Greaney ML, De Jesus M, Sprunck-Harrild KM, et al. Peer Reviewed: Designing audience-centered interactive voice response messages to promote cancer screenings among low-income Latinas. *Prev Chronic Dis*. 2014;**11**:E40.

* 59. Pietrzak P, Godlewski D, Adamczak M. Breast cancer screening program attitudes among women from Wielkopolska. The report from Focus Group Interview (FGI) studies. *Wspolczesna Onkologia-Contemporary Oncology*. 2011;**15**:415-416.

* 60. Oscarsson MG, Wijma BE, Benzein EG. 'I do not need to... I do not want to... I do not give it priority...' - Why women choose not to attend cervical cancer screening. *Health Expect*. 2008;**11**:26-34.

61. Garber JE, Offit K. Hereditary cancer predisposition syndromes. *J Clin Oncol*. 2005;**23**:276-292.

* 62. Kahn JA. Beliefs about Papanicolaou smears and compliance with Papanicolaou smear follow-up in adolescents. *Arch Pediatr Adolesc Med*. 1999;**153**:1046-1054.

* 63. Ndikom CM, Ofi BA. Awareness, perception and factors affecting utilization of cervical cancer screening services among women in Ibadan, Nigeria: a qualitative study. *Reprod Health*. 2012;**9**:11.

* 64. Brouse CH, Basch CE, Wolf RL, Shmukler C, Neugut AI, Shea S. Barriers to colorectal cancer screening with fecal occult blood testing in a predominantly minority urban population: a qualitative study. *Am J Public Health*. 2003;**93**:1268-1271.

* 65. Todd A, Stuifbergen A. Barriers and facilitators to breast cancer screening: a qualitative study of women with multiple sclerosis. *Int J MS Care*. 2011;**13**:49-56.

* 66. Kwok C, Cant R, Sullivan G. Factors associated with mammographic decisions of Chinese-Australian women. *Health Educ Res*. 2005;**20**:739-747.

67. Austin LT, Ahmad F, McNally M-J, Stewart DE. Breast and cervical cancer screening in Hispanic women: a literature review using the health belief model. *Womens Health Issues*. 2002;**12**:122-128.

68. Jack R, Davies E, Møller H. Breast cancer incidence, stage, treatment and survival in ethnic groups in South East England. *Br J Cancer*. 2009;**100**:545-550.

69. Gathani T, Ali R, Balkwill A, et al. Ethnic differences in breast cancer incidence in England are due to differences in known risk factors for the disease: prospective study. *Br J Cancer*. 2014;**110**:224-229.

70. Chlebowski RT, Chen Z, Anderson GL, et al. Ethnicity and breast cancer: factors influencing differences in incidence and outcome. *J Natl Cancer Inst*. 2005;**97**:439-448.

71. Szczepura A, Price C, Gumber A. Breast and bowel cancer screening uptake patterns over 15 years for UK south Asian ethnic minority populations, corrected for differences in socio-demographic characteristics. *BMC Public Health*. 2008;**8**:346.

72. Hoare T. Breast screening and ethnic minorities. *Br J Cancer*. 1996;**29(Suppl.)**: S38-41.

73. Cooper GS, Koroukian SM. Racial disparities in the use of and indications for colorectal procedures in Medicare beneficiaries. *Cancer*. 2004;**100**:418-424.

74. Irby K, Anderson WF, Henson DE, Devesa SS. Emerging and widening colorectal carcinoma disparities between Blacks and Whites in the United States (1975-2002). *Cancer Epidemiol Biomarkers Prev*. 2006;**15**:792-797.

75. Chien C, Morimoto LM, Tom J, Li CI. Differences in colorectal carcinoma stage and survival by race and ethnicity. *Cancer*. 2005;**104:**629-639.

76. Consedine NS, Magai C, Krivoshekova YS, Ryzewicz L, Neugut AI. Fear, anxiety, worry, and breast cancer screening behavior: a critical review. *Cancer Epidemiol Biomarkers Prev*. 2004;**13**:501-510.

77. Balmer C, Griffiths F, Dunn J. A qualitative systematic review exploring lay understanding of cancer by adults without a cancer diagnosis. *J Adv Nurs*. 2014;**70**:1688-1701.

* 78. Kawar LN. Barriers to breast cancer screening participation among Jordanian and Palestinian American women. *Eur J Oncol Nurs*. 2013;**17**:88-94.

* 79. Holmes-Rovner M, Williams GA, Hoppough S, Quillan L, Butler R, Given CW. Colorectal cancer screening barriers in persons with low income. *Cancer Pract*. 2002;**10**:240-247.

80. Hollada J, Speier W, Oshiro T, et al. Patients' perceptions of radiation exposure associated with mammography. *AJR Am J Roentgenol*. 2015;**205:**215-221.

* 81. Griffiths F, Bendelow G, Green E, Palmer J. Screening for breast cancer: medicalization, visualization and the embodied experience. *Health (London)*. 2010;**14**:653-668.

* 82. Kahn LS, Fox CH, Krause-Kelly J, Berdine DE, Cadzow RB. Identifying barriers and facilitating factors to improve screening mammography rates in women diagnosed with mental illness and substance use disorders. *Women Health*. 2005;**42**:111-126.

* 83. Kwok C, Sullivan G, Cant R. The role of culture in breast health practices among Chinese-Australian women. *Patient Educ Couns*. 2006;**64**:268-276.

* 84. Jepson RG, Hewison J, Thompson A, Weller D. Patient perspectives on information and choice in cancer screening: a qualitative study in the UK. *Soc Sci Med*. 2007;**65**:890-899.

* 85. Engelman KK, Cizik AM, Ellerbeck EF, Rempusheski VF. Perceptions of the screening mammography experience by Hispanic and non-Hispanic White women. *Womens Health Issues*. 2012;**22**:e395-e401.

* 86. Azaiza F, Cohen M. Between traditional and modern perceptions of breast and cervical cancer screenings: a qualitative study of Arab women in Israel. *Psychooncology*. 2008;**17**:34-41.

* 87. Teng FF, Mitchell SM, Sekikubo M, et al. Understanding the role of embarrassment in gynaecological screening: a qualitative study from the ASPIRE cervical cancer screening project in Uganda. *BMJ Open*. 2014;**4**:e004783.

* 88. Wittenberg E, Bharel M, Saada A, Santiago E, Bridges JF, Weinreb L. Measuring the preferences of homeless women for cervical cancer screening interventions: development of a best–worst scaling survey. *Patient*. 2015;**8**:455-467.

* 89. Busingye P, Nakimuli A, Nabunya E, Mutyaba T. Acceptability of cervical cancer screening via visual inspection with acetic acid or Lugol's iodine at Mulago Hospital, Uganda. *Int J Gynaecol Obstet*. 2012;**119**:262-265.

* 90. Jones RM, Devers KJ, Kuzel AJ, Woolf SH. Patient-reported barriers to colorectal cancer screening: a mixed-methods analysis. *Am J Prev Med*. 2010;**38**:508-516.

* 91. Logan L, McIlfatrick S. Exploring women's knowledge, experiences and perceptions of cervical cancer screening in an area of social deprivation. *Eur J Cancer Care (Engl)*. 2011;**20**:720-727.

* 92. O'Brien BA, Mill J, Wilson T. Cervical screening in Canadian First Nation Cree women. *J Transcult Nurs*. 2009;**20**:83-92.

* 93. Truesdale-Kennedy M, Taggart L, McIlfatrick S. Breast cancer knowledge among women with intellectual disabilities and their experiences of receiving breast mammography. *J Adv Nurs*. 2011;**67**: 1294-1304.

94. Balasooriya-Smeekens C, Walter FM, Scott S. The role of emotions in time to presentation for symptoms suggestive of cancer: a systematic literature review of quantitative studies. *Psychooncology*. 2015;**24**:1594-1604.

95. Robb K, Stubbings S, Ramirez A, et al. Public awareness of cancer in Britain: a population-based survey of adults. *Br J Cancer*. 2009;**101(Suppl.2)**:S18-S23.

* 96. Best AL, Spencer M, Hall IJ, Friedman DB, Billings D. Developing spiritually framed breast cancer screening messages in consultation with African American women. *Health Commun*. 2015;**30**:290-300.

* 97. Kelly KM, Schoenberg N, Wilson TD, Atkins E, Dickinson S, Paskett E. Cervical cancer worry and screening among Appalachian women. *J Prim Prev*. 2015;**36**:79-92.

* 98. Granado MN, Guell C, Hambleton IR, Hennis AJ, Rose AM. Exploring breast cancer screening barriers among Barbadian women: a focus group study of mammography in a resource-constrained setting. *Critical Public Health*. 2014;**24**:429-444.

* 99. Opoku SY, Benwell M, Yarney J. Knowledge, attitudes, beliefs, behaviour and breast cancer screening practices in Ghana, West Africa. *Pan Afr Med J*. 2012;**11**:28.

100. Franklin MD, Schlundt DG, McClellan L, et al. Religious fatalism and its association with health behaviors and outcomes. *Am J Health Behav*. 2007;**31**:563-572.

* 101. Green AR, Peters-Lewis A, Percac-Lima S, et al. Barriers to screening colonoscopy for low-income Latino and white patients in an urban community health center. *J Gen Intern Med*. 2008;**23**:834-840.

* 102. Woodrow C, Watson E, Rozmovits L, Parker R, Austoker J. Public perceptions of communicating information about bowel cancer screening. *Health Expect*. 2008;**11**:16-25.

* 103. Dolezil D, Haase A, Jahnke K, et al. Motives and attitudes of the elderly towards cancer screening: an explorative mixed-methods study. *Eur J Gen Pract*. 2014;**20**:219-228.

* 104. Austin KL, Power E, Solarin I, Atkin WS, Wardle J, Robb KA. Perceived barriers to flexible sigmoidoscopy screening for colorectal cancer among UK ethnic minority groups: a qualitative study. *J Med Screen*. 2009;**16**:174-179.

* 105. Bass SB, Gordon TF, Ruzek SB, et al. Perceptions of colorectal cancer screening in urban African American clinic patients: differences by gender and screening status. *J Cancer Educ*. 2011;**26**:121-128.

* 106. Lee YY, Jun JK, Suh M, Park B, Kim Y, Choi KS. Barriers to cancer screening among medical aid program recipients in the Republic of Korea: a qualitative study. *Asian Pac J Cancer Prev*. 2013;**15**:589-594.

* 107. Adejimi AA, Akinleye CA. Knowledge, attitude and practices of cervical cancer prevention among women attending HIV treatment centre in Lautech Teaching Hospital, Nigeria: a qualitative study. *Asia Pac J Clin Oncol*. 2014;**10**:189-189.

* 108. Paz-Soldan VA, Nussbaum L, Bayer AM, Cabrera L. Low knowledge of cervical cancer and cervical pap smears among women in Peru, and their ideas of how this could be improved. *Int Q Community Health Educ*. 2010-2011;**31**:245-263.

* 109. Lyttle NL, Stadelman K. Assessing awareness and knowledge of breast and cervical cancer among Appalachian women. *Prev Chronic Dis*. 2006;**3**:A125.

* 110. Keshavarz Z. Effective factors on performing breast and cervix cancer screening test by Iranian workers: A qualitative-model based study. *Int J Gynaecol Obstet*. 2012;**119**:S854.

* 111. Filippi MK, James AS, Brokenleg S, et al. Views, barriers, and suggestions for colorectal cancer screening among american Indian women older than 50 years in the midwest. *J Prim Care Community Health*. 2013;**4**:160-166.

* 112. Denizard-Thompson NM, Weaver KE, Ellis SD, Kronner D, Miller DP. Older adults' attitudes about receiving text messages related to colorectal cancer screening. *J Gen Intern Med.* 2014;**29**:S157.

* 113. Cadman L, Waller J, Ashdown-Barr L, Szarewski A. Barriers to cervical screening in women who have experienced sexual abuse: an exploratory study. *J Fam Plann Reprod Health Care.* 2012;**38**:214-220.

* 114. Cadman L, Ashdown-Barr L, Waller J, Szarewski A. Attitudes towards cytology and human papillomavirus self-sample collection for cervical screening among Hindu women in London, UK: a mixed methods study. *J Fam Plann Reprod Health Care*. 2015;**41**:38-47.

* 115. Jilcott Pitts SB, Lea CS, May CL, et al. "Fault-line of an Earthquake": a qualitative examination of barriers and facilitators to colorectal cancer screening in rural, Eastern North Carolina. *J Rural Health*. 2013;**29**:78-87.

116. Robb KA, Power E, Atkin W, Wardle J. Ethnic differences in participation in flexible sigmoidoscopy screening in the UK. *J Med Screen*. 2008;**15**:130-136.

117. Wardle J, Miles A, Atkin W. Gender differences in utilization of colorectal cancer screening. *J Med Screen*. 2005;**12**:20-27.

* 118. Schoenberg NE, Hopenhayn C, Christian A, Knight EA, Rubio A. An in-depth and updated perspective on determinants of cervical cancer screening among central Appalachian women. *Women Health*. 2005;**42**:89-105.

119. Niksic M, Rachet B, Warburton FG, Wardle J, Ramirez AJ, Forbes LJ. Cancer symptom awareness and barriers to symptomatic presentation in England-are we clear on cancer? *Br J Cancer*. 2015;**113**:533-542.

* 120. Saadi A, Bond B, Percac-Lima S. Perspectives on preventive health care and barriers to breast cancer screening among Iraqi women refugees. *J Immigr Minor Health*. 2012;**14**:633-639.

* 121. Friedman AM, Hemler JR, Rossetti E, Clemow LP, Ferrante JM. Obese women's barriers to mammography and pap smear: the possible role of personality. *Obesity*. 2012;**20**:1611-1617.

122. Kwok C, Sullivan G. Chinese-Australian women's beliefs about cancer: implications for health promotion. *Cancer Nursing*. 2006;**29**:E14-E21.

* 123. Nolan J, Renderos TB, Hynson J, et al. Barriers to cervical cancer screening and follow-up care among Black women in Massachusetts. *J Obstet Gynecol Neonatal Nurs*. 2014;**43**:580-588.

* 124. Frisby CM. Messages of hope: Health communication strategies that address barriers preventing Black women from screening for breast cancer. *J Black Stud*. 2002;**32**:489-505.

125. Lannin DR, Mathews HF, Mitchell J, Swanson MS, Swanson FH, Edwards MS. Influence of socioeconomic and cultural factors on racial differences in late-stage presentation of breast cancer. *JAMA*. 1998;**279**:1801-1807.

126. Jones CE, Maben J, Jack RH, et al. A systematic review of barriers to early presentation and diagnosis with breast cancer among black women. *BMJ Open*. 2014;**4**:e004076.

127. O'Neill KM, Mandigo M, Pyda J, et al. Out-of-pocket expenses incurred by patients obtaining free breast cancer care in Haiti. *Lancet*. 2015;**385(Suppl 2)**:S48.

128. Ramsey S, Blough D, Kirchhoff A, et al. Washington State cancer patients found to be at greater risk for bankruptcy than people without a cancer diagnosis. *Health affairs*. 2013;**32**:1143-1152.

* 129. Goldsmith G, Chiaro C. Colorectal cancer screening: how to help patients comply. *J Fam Pract*. 2008;**57**:E2-7.
